# RNA-Seq Analysis of Abdominal Fat in Genetically Fat and Lean Chickens Highlights a Divergence in Expression of Genes Controlling Adiposity, Hemostasis, and Lipid Metabolism

**DOI:** 10.1371/journal.pone.0139549

**Published:** 2015-10-07

**Authors:** Christopher W. Resnyk, Chuming Chen, Hongzhan Huang, Cathy H. Wu, Jean Simon, Elisabeth Le Bihan-Duval, Michel J. Duclos, Larry A. Cogburn

**Affiliations:** 1 Department of Animal and Food Sciences, University of Delaware, Newark, Delaware, United States of America; 2 Center for Bioinformatics and Computational Biology, University of Delaware, Newark, Delaware, United States of America; 3 INRA UR83 Recherches Avicoles, 37380, Nouzilly, France; Seoul National University, REPUBLIC OF KOREA

## Abstract

Genetic selection for enhanced growth rate in meat-type chickens (*Gallus domesticus*) is usually accompanied by excessive adiposity, which has negative impacts on both feed efficiency and carcass quality. Enhanced visceral fatness and several unique features of avian metabolism (i.e., fasting hyperglycemia and insulin insensitivity) mimic overt symptoms of obesity and related metabolic disorders in humans. Elucidation of the genetic and endocrine factors that contribute to excessive visceral fatness in chickens could also advance our understanding of human metabolic diseases. Here, RNA sequencing was used to examine differential gene expression in abdominal fat of genetically fat and lean chickens, which exhibit a 2.8-fold divergence in visceral fatness at 7 wk. Ingenuity Pathway Analysis revealed that many of 1687 differentially expressed genes are associated with hemostasis, endocrine function and metabolic syndrome in mammals. Among the highest expressed genes in abdominal fat, across both genotypes, were 25 differentially expressed genes associated with *de novo* synthesis and metabolism of lipids. Over-expression of numerous adipogenic and lipogenic genes in the FL chickens suggests that *in situ* lipogenesis in chickens could make a more substantial contribution to expansion of visceral fat mass than previously recognized. Distinguishing features of the abdominal fat transcriptome in lean chickens were high abundance of multiple hemostatic and vasoactive factors, transporters, and ectopic expression of several hormones/receptors, which could control local vasomotor tone and proteolytic processing of adipokines, hemostatic factors and novel endocrine factors. Over-expression of several thrombogenic genes in abdominal fat of lean chickens is quite opposite to the pro-thrombotic state found in obese humans. Clearly, divergent genetic selection for an extreme (2.5–2.8-fold) difference in visceral fatness provokes a number of novel regulatory responses that govern growth and metabolism of visceral fat in this unique avian model of juvenile-onset obesity and glucose-insulin imbalance.

## Introduction

The domestic chicken (*Gallus domesticus*) serves a dual purpose as a world-wide source of high-quality dietary protein and as an important model organism for developmental biology and genomics research [1–4]. In particular, the chicken has been used extensively as a biomedical model to understand basic mechanisms controlling embryonic development, immune system function, nutrient utilization, hormone sensitivity, and adiposity. Chickens have several unique metabolic attributes which make them an attractive model for obesity related studies. Unlike mammals, chickens naturally exhibit hyperglycemia (>200 mg/dL during fasting) and survive large doses of exogenous insulin, indicating an innate insensitivity to insulin, particularly in adipose tissue where insulin exerts only marginal effects on the uptake of glucose by isolated adipocytes [5,6]. Another unique feature of metabolic regulation in the chicken is the disruption of syntenic genomic loci of five major mammalian adipokines [leptin (*LEP*) [7], plasminogen activator inhibitor–1 (*PAI–1*), tissue necrosis factor alpha (*TNFA*), resistin and omentin [8]]. The absence of these key adipokines in the chicken, particularly *LEP* [9], indicates that alternative mechanisms must exist to regulate their feed intake and the balance between energy expenditure and storage. Despite a major class difference in absence/presence of adipokines, chickens do share several key metabolic characteristics with humans, including the fact that the liver is the primary site of *de novo* synthesis of lipids [10–13], which are then transported as triglycerides to adipose tissue for storage and release. In chickens, abdominal fatness is a highly-heritable polygenic trait regulated by multiple behavioral, environmental and hormonal factors [14–21].

Recent high-density microarray studies have shown that lipogenic genes are readily transcribed in chicken adipose tissue [22,23] and developmentally regulated in genetically fat (FL) and lean (LL) chickens [24]. Using a combined metabolomics and transcriptomic approach, Ji *et al*. [23] compared gene expression and metabolite profiles in abdominal fat of relatively leaner-chicken breeds (Leghorn and Fayoumi) against fatter and heavier commercial broiler chickens at the same age (7 wk). Their main conclusions were that abdominal leanness in the Leghorn and Fayoumi breeds was achieved by enhanced lipid catabolism and reduced lipid synthesis in abdominal fat, whereas enhanced adipogenesis and greater fatness in broiler chickens reflect a reduction in both fatty-acid oxidation and liberation of non-esterified fatty acids by visceral fat. In contrast, the FL and LL chickens used in the present study were divergently selected over seven generations for either high (FL) or low (LL) abdominal fatness at similar body weights and feed intake [25]. These unique FL and LL chickens serve as valid genetic models [26–28] of leanness and juvenile-onset obesity with a 2.5-fold difference in abdominal fatness between 3 to 11 weeks of age (wk) [25,29]. Our recent time-course microarray analysis of abdominal fat in FL and LL chickens [24] has revealed numerous differentially expressed (DE) genes involved in several processes, which ultimately produce either a lipolytic (LL) or lipogenic (FL) state. Among the DE genes, we found extensive overexpression of endocrine, hemostatic, lipolytic, and lipid export genes in the diminished abdominal fat of LL cockerels, especially at 7 wk. On the other hand, visceral fat of the FL chickens show high expression of multiple transcription factors, enzymes and transporters involved in adipogenesis and lipogenesis [24]. In another transcriptional study of adipose tissue in juvenile chickens of a commercial broiler cross, short-term fasting (5 h) resulted in altered expression of 1780 genes, while acute insulin immunoneutralization affected only 92 adipose genes [22]. This relatively short period of fasting was sufficient to down regulate adipose genes associated with synthesis, elongation and desaturation of fatty acids. However, a gap remains in our understanding of adipogenesis in chickens, especially the importance of *de novo* synthesis of lipids in visceral adipose tissue and the function of numerous endocrine factors/receptors expressed by abdominal fat.

For more than three decades, scores of papers have described various aspects of growth and nutrient metabolism in the divergently-selected FL and LL chickens originally developed by Leclerq *et al* [25]. In general, the FL and LL cockerels have similar growth rates with a 2.5-fold difference in abdominal fatness and higher breast muscle weights in the LL. The FL chickens always exhibit a lower plasma glucose level without overt hyperinsulinemia found in mammals, a peculiar condition which Simon *et al*. [30,31] described as a “glucose-insulin imbalance”. Hypertriglyceridemia of the FL chickens indicates higher rate of hepatic lipogenesis from carbohydrate metabolism, most likely the consequence of a small increase in insulin-sensitivity in the FL chickens [26,31]. The FL and LL chickens are able to maintain their respective fat or lean phenotype independently of altered energy sources [32], emphasizing genetic regulation of phenotypic expression. These metabolic peculiarities in our polygenic model of juvenile-onset obesity have been extensively examined by nutritional and metabolic perturbations [26,33–36]. Furthermore, transcriptional profiling of multiple tissues [2,18,24,37–41] and high-throughput surveys of variations in genome sequence and structure, including extensive quantitative trait loci (QTL) and expression (eQTL) analyses [17,19,20,42–44] in the FL and LL chickens have begun to identify causal genes and to unravel the genetic and molecular basis for their divergence in lipid metabolism and visceral fatness.

The present descriptive transcriptomics study, using RNA sequencing (RNA-Seq) analysis, was designed to expand our catalog of expressed adipose genes at 7 wk with a dual goal (1) to determine the most transcriptionally-active biological processes in abdominal fat and (2) to establish major functional differences between the abdominal fat transcriptomes of FL and LL chickens. First, a functional characterization of the 900 highest expressed (HE) adipose genes independent of genotype was provided by a bioinformatics analysis. Second, we identified 1687 differentially-expressed (DE) genes from the comparison of transcripts in abdominal fat of FL and LL chickens. Ingenuity Pathway Analysis has revealed the over-expression of numerous genes involved in hemostasis, lipid catabolism, and endocrine signaling in the LL. In contrast, the up-regulation of several key adipogenic and lipogenic genes in abdominal fat of the FL chickens suggests that *in situ* lipogenesis could make a more substantial contribution to the expansion of adipose mass in the chicken than previously recognized.

## Materials and Methods

### Animals and tissue preparation

The FL and LL chickens were bred and raised at INRA UE1295 Pôle d'Expérimentation Avicole de Tours, F–37380 Nouzilly, France, as described previously [24]. Briefly, 8 birds from each genotype (FL and LL) were randomly selected for tissue sampling at six ages (1, 3, 5, 7, 9, and 11 wk), weighed, bled into heparinized syringes, and killed by cervical dislocation. Abdominal fat was quickly dissected, weighed, a sample was immediately snap frozen in liquid nitrogen, and stored at −75°C for further processing. All animal procedures were performed under the strict supervision of a French government veterinarian and in accordance with protocols approved by the French Agricultural Agency, the Scientific Research Agency, and the Institutional Animal Care and Use Committee at INRA, Nouzilly, France. These procedures were also in compliance with the United States Department of Agriculture guidelines on the use of agricultural animals in research and approved by the University of Delaware Agricultural Animal Care and Use Committee.

### RNA extraction, library preparation and RNA sequencing

Abdominal fat samples from eight individual 7-wk-old chickens (4 FL and 4 LL) were homogenized and cellular RNA extracted using guanidine thiocyanate and CsCl gradient purification [45] followed by DNase I treatment. Sample quality was analyzed with an RNA 6000 Nano Assay kit and the Model 2100 Bioanalyzer (Agilent Technologies; Palo Alto, CA). The rRNA ratio (28S/18S) was determined and all samples had an RNA integrity number (RIN) greater than 9.0. Sequencing libraries were made from 1 μg of total adipose RNA with the Illumina RNA Sample Prep Kit v2 following standard Illumina protocols. Individual RNA samples were indexed (bar-coded) to enable multiplexing of libraries within sequencing lanes. Libraries were pooled and sequenced using an Illumina HiSeq 2000 Sequencing System at the Delaware Biotechnology Institute, University of Delaware. Three separate schemes were used for paired-end (101 bp) sequencing of 8 libraries (4 FL and 4 LL) across two sequencing lanes per run. In Scheme A, two sequencing lanes were used for multiplexing of two FL and two LL samples per lane (n = 4/lane). Two libraries (1 FL and 1 LL) in sequencing lane 2 of Scheme A had low quality control (QC) scores and were eliminated from further analyses. Consequently, the two low QC libraries were re-sequenced in individual lanes in Scheme B (n = 1/lane). Finally, all eight (4 FL and 4 LL) libraries were multiplexed and sequenced in two replicate lanes in Scheme C (n = 8/lane). All samples in Schemes 1–3 were merged into one file of 12 samples from the FL and 12 samples from the LL cockerels for further analysis.

### RNA sequence (RNA-seq) analysis

All reads generated from the three sequencing schemes (12 FL and 12 LL) described above were included in the RNA-Seq analysis using CLC Genomics Workbench 5.1 software (CLC bio, Cambridge, MA). The data analysis included sequence data filtering, read mapping, transcript and gene identification, analysis of differential gene expression, and functional annotation.

#### Sequence data filtering

Twenty-four short-read (101 base pairs) sequencing samples (12 FL and 12 LL) from the 3 sequencing schemes were de-multiplexed and imported into CLC Genomics Workbench, separately. Several QC trimming methods were used within the CLC Genomics Workbench software, including quality trimming, ambiguity trimming and adapter trimming with default settings applied before mapping to the reference chicken genome.

#### Read mapping and transcript/gene identification

The reference genome for the chicken (*Gallus gallus*, build 2.1) in FASTA format and the corresponding annotation file in GTF format were obtained from Ensembl (ftp.ensembl.org/pub/release–64), which represents 17,934 genes and 22,298 transcripts. Two hundred nucleotides of flanking region upstream and downstream of known genes were also included in the analysis. The short paired-end read sequences (101 bp x 2) were mapped to the reference chicken genome sequence, with mapping parameters that enforced: (1) a maximum of two mismatches and (2) reads must map with ≥ 90% of the bases aligned to the reference sequence with ≥ 80% similarity. Non-specific matches (reads mapped to multiple places in the reference genome) were excluded from the analysis.

#### Differential expression analysis

The unique exon reads count (including the exon-exon and exon-intron junctions) for the reads mapped to a gene and its flanking regions were used as the raw expression value for that gene. This raw expression value was normalized to the median of the total mapped reads across the 24 samples to account for variation in original library concentration and multiplexing number. The 24 sequencing samples were divided into two genotypes (FL and LL), resulting in 12 replicates for each genotype. Normalized expression values were analyzed as a beta-binomial model [46] to detect differential expression. The two-sided P-value was corrected using the false discovery rate (FDR) adjustment to account for multiple hypothesis testing procedures [47]. Genes with FDR-adjusted P-value (*≤*0.05) were considered to be statistically significant. To ensure the biological relevance, a condition of fold change ≥ 1.2 (or ≤ -1.2) was added on top of FDR-adjusted P-value (*≤*0.05); Genes with FDR-adjusted P-value (*≤*0.05) and fold change ≥ 1.2 (or ≤ -1.2) were considered to be differentially expressed (DE) in this study. The fold-change threshold (±1.2-fold) for DE genes is based on our extensive experience in functional genomics and transcriptional profiling of multiple tissues from various chicken models. A recent RNA-Seq study of breast muscle in chickens afflicted with “Wooden Breast” disease [48] used a similar significance level [FDR-adjusted P-value (*≤*0.05) and ±1.3 fold-change] to identify DE genes using default settings for the Cuffdiff procedure in the open-source software, Cufflinks (http://cole-trapnell-lab.github.io/cufflinks/).

### Availability of supporting data

The RNA-Seq reads in Sequence Read Archive (SRA) format were deposited into the National Center for Biotechnology Information Gene Expression Omnibus (NCBI GEO) database under the accession # GSE42980. Further data sets supporting the present results are included within the article and in supporting information files.

### Quantitative RT-PCR analysis

For verification of expression, quantitative real-time PCR (qRT-PCR) analysis was performed on a subset of 47 DE genes identified by RNA-Seq analysis. First-strand cDNA synthesis was performed by incubation of a 13 μl reaction (containing 1 μg of total DNase-treated RNA, 1 μl of 100 μM oligo dT_20_, 1 μl of 10 mM dNTP mix, and water to 13 μl total volume) for 5 min at 70°C and placed on ice for 2 min. A master mix containing 5 μl of 5x first-strand synthesis buffer, 1 μl of 0.1 M DTT, 1 μl of RNaseOUT, and 200 U of SuperScript III reverse transcriptase (Invitrogen, Carlsbad, CA) was added (final reaction volume of 20 μl). Primers were designed for qRT-PCR using Primer Express v2.0 software (Applied Biosystems, Foster City, CA). Detailed information for each primer pair, including gene name, gene symbol, forward and reverse primer sequences, GenBank accession number and amplicon size, are provided in S1 Table.

An ABI Prism Sequence Detection System 7900HT was used to perform the qRT-PCR assays, using 10 ng of total RNA, Power SYBR green PCR master mix (Applied Biosystems, Foster City, CA), and 400 nM of each primer pair (Sigma-Aldrich, St. Louis, MO) in duplicate wells. A disassociation step was used to validate specific amplification and verify absence of primer dimers. PCR products were analyzed using agarose gel electrophoresis to compare product size to the expected amplicon size. The cycle time (Ct) for each sample was normalized to the corresponding sample geometric mean of housekeeping genes [49]. We selected two housekeeping genes [pantothenate kinase 1 (*PANK1*) and ribosomal protein L14 (*RPL14*)] based on their invariability in qRT-PCR analysis and identified as the most stably-expressed genes using RefFinder software (http://www.leonxie.com/referencegene.php). The 2^-(ΔΔCt)^ formula was used to calculate relative abundance of transcripts [50]. The statistical analysis of normalized qRT-PCR data across three ages (3, 7 and 11 wk) was performed using a general linear model (GLM) procedure in Statistical Analysis System (SAS v9.3; Cary, NC). These data were analyzed using a two-factor analysis of variance to determine main effects (*P*≤0.05) of genotype (G), age (A), and the interaction of age with genotype (A × G). Where genes were only used for qRT-PCR analysis at one age (7 wk), a student’s T-test was used to identify significant (*P*≤0.05) differences between the FL and LL genotypes.

## Results

### Mapped reads and detection of genes and transcripts

Sequence data from the 24 samples were mapped to the reference genome (*Gallus gallus*, build 2.1). Table 1 presents a summary of the RNA-Seq analysis including the number of mapped reads and detection of corresponding chicken genes and transcripts (see S2 Table for more details). The original sequencing run (Scheme A) was completed by multiplexing 2 FL and 2 LL samples (N = 4) in two separate sequencing lanes. Two samples (1 FL and 1 LL) in Lane 2 had low quality scores; therefore, the low-quality data was eliminated from further RNA-Seq analysis. Nonetheless, Scheme A provided an average of 32.5 M mapped reads for the FL (N = 3) and 36 M mapped reads for the LL (N = 3), which allowed detection of 73% genes and 65% of transcripts across the FL and LL genotypes. Subsequently, the two low quality score libraries were re-sequenced in separate lanes in another sequencing run (Scheme B), which gave the highest average detection levels of 78% for genes and 70% for transcripts across both genotypes. In Scheme C, the 8 libraries (4 FL and 4 LL samples) were multiplexed and sequenced in duplicate lanes within the same sequencing run. Scheme C represents the balanced block design with two technical replicates as described by Auer and Doerge [51] for proper statistical analysis of RNA-Seq experiments. Comparing sequencing depths (averaged across the FL and LL chickens), Scheme C allowed detection of 71% of genes and 63% of transcripts by multiplexing 8 libraries per lane which were sequenced in duplicate lanes. The average number of reads mapped across Scheme A, B and C was greater for the LL (41.6 M) than the FL (35.4 M), which is reflected in the slightly higher number of expressed genes found in abdominal fat of the LL cockerels. The overall average across genotype (FL and LL) and sequencing schemes (A, B and C) shows that 45% (38.5 M) of the total reads were mapped, which equates to identification of 74% of genes (13,265/17,934) and 66% of transcripts (14,724/22,298) based on the reference chicken genome. Genes from the reference chicken genome were mapped to UniProtKB accession numbers by the Protein Information Resource (PIR) ID mapping service [52]. The assigned fold-change values (i.e., FL/LL expression ratios) were based on the number of normalized reads from the RNA-Seq analysis.

**Table 1:** Summary of RNA-Seq analysis of abdominal fat in divergent FL and LL chickens at 7 wk.

Scheme (samples/lane)	Total input reads	Paired-end reads after trimming	Total reads mapped	Total reads unmapped	Expressed Genes	Expressed Transcripts
**A (n = 3)** * **: FL**	59.13M	58.71M	32.54M	26.59M	12,959	14,358
**A (n = 3)** * **: LL**	67.11M	27.54M	36.02M	31.09M	13,187	14,599
**B (n = 1): FL**	123.68M	122.2M	54.64M	69.05M	13,890	15,550
**B (n = 1): LL**	187.85M	182.7M	71.05M	116.8M	14,134	15,853
**C (n = 8): FL**	40.26M	34.22M	19.12M	21.14M	12,810	14,081
**C (n = 8): LL**	35.71M	35.34M	17.67M	18.04M	12,612	13,902
**Average Across Schemes: FL**	74.36M	71.71M	35.43M	38.92M	13,220	14,663
**Average Across Schemes: LL**	96.89M	81.86M	41.58M	55.31M	13,311	14,785
**Average Across Genotypes and schemes (A,B,C)**	85.62M	76.78M	38.51M	47.12M	13,265	14,724

Read trimming, read mapping and expression data are provided for three different sequencing schemes utilized for paired-end sequencing of 8 libraries (4 FL and 4 LL cockerels). Values are averaged across 3–4 individual birds per genotype. In Scheme A, two sequencing lanes were used for assignment of two FL and two LL libraries per lane (n = 4/lane).

*Two libraries (1 FL and 1 LL) in sequencing lane 2 (Scheme A) had a low QC score; therefore, their low-quality data were eliminated from further RNA-Seq analysis under this scheme. Consequently, these two libraries were each re-sequenced in individual lanes in Scheme B (n = 1/lane). Finally, all eight libraries (4 FL and 4 LL) were sequenced in two replicate lanes in Scheme C (n = 8/lane). Scheme C provides the most robust RNA-Seq design, the balanced block design; where, all eight libraries (4 FL and 4 LL) are sequenced in two replicate sequencing lanes. This design allows the targeted biological variation to be partitioned from technical error as described in detail by Auer and Doerge [51]. Additional information on RNA-Seq analysis is provided in S2 Table.

*Abbreviations*: fat line (FL), lean line (LL), and million (M).

A power analysis was performed on this RNA-Seq dataset using the web-based software program “Scotty” (http://euler.bc.edu/marthlab/scotty/scotty.php) [53]. This analysis demonstrates that our sample size of four birds/genotype (n = 4), sequenced across three depths [38.5 million mapped reads per sample averaged across depths (Table 1)], had power to detect 80% of genes expressed in each genotype (*P*≤0.01) at ≥ 1.5 fold difference and greater than 90% at a fold-change of ≥ 2 (S1 Fig). Further, the “Scotty” program performed hierarchical clustering using Spearman correlation as the distance metric. This correlation analysis grouped the two genotypes distinctly, where the individuals within each genotype (FL and LL) were closely linked.

### Abdominal fat transcriptome of fed FL and LL chickens

**Fig 1:**
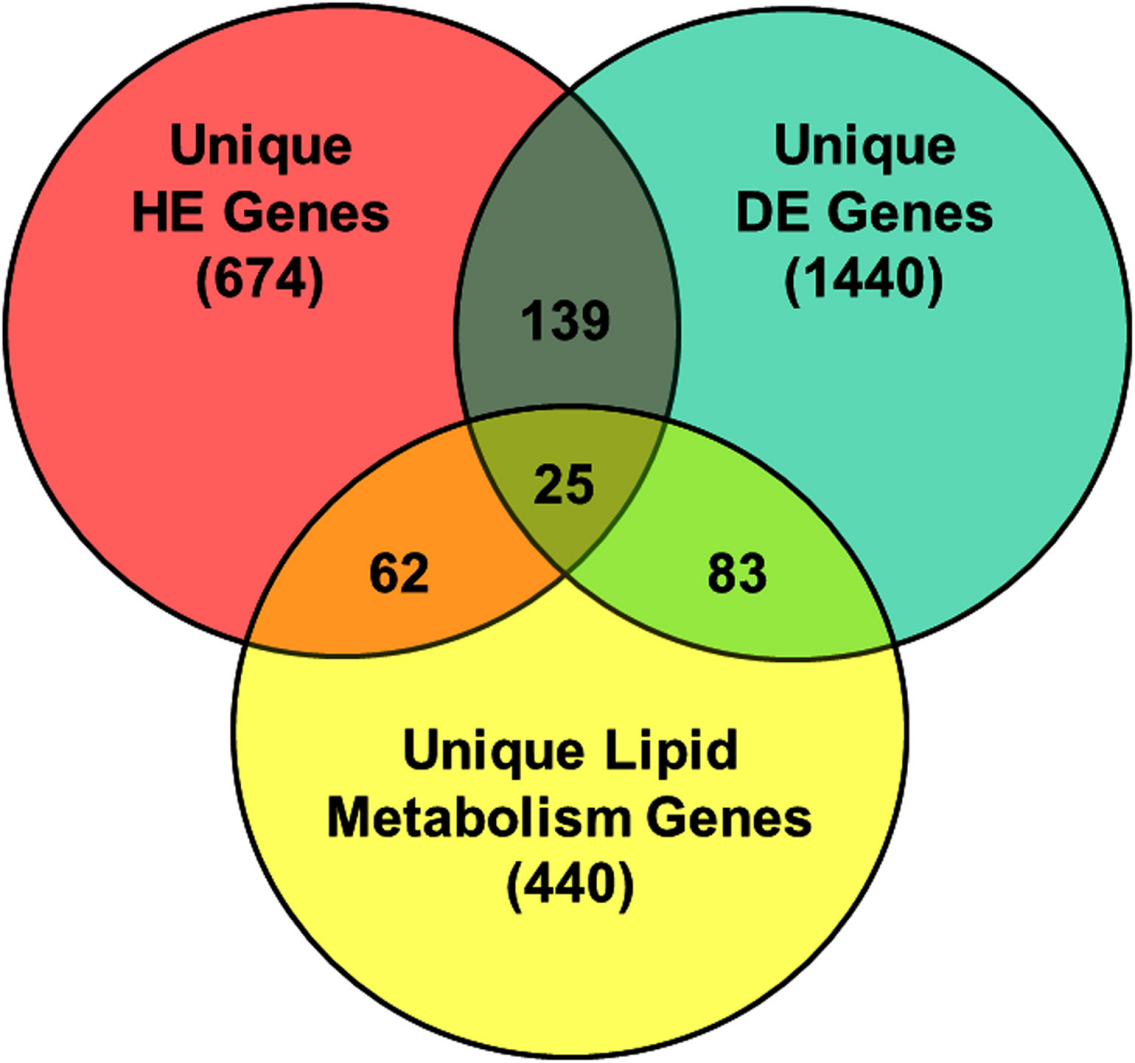
Venn diagram illustrating overlap among functional gene lists. The Venn diagram shows the intersections of highly expressed (HE) genes in abdominal fat (S3 Table), differentially expressed (DE) genes (S4 Table), and Ingenuity annotated genes known to be involved in lipid metabolism (S5 Table). The numbers in overlapping arcs indicate the number of genes shared between and among these three categories.

First, the 900 highest-expressed (HE) genes, defined as genes with an average (across both genotypes) of >4289 reads/gene, were identified from RNA-Seq analysis of abdominal fat in FL and LL chickens at 7 wk (S3 Table). Of the HE genes, 164 were expressed higher in the FL (>1.2-fold difference), while 155 HE genes were up-regulated (< -1.2-fold difference) in visceral fat of the LL. Second, we identified 1687 DE genes with a FDR-corrected P-value (*P*≤0.05) and fold change ≥ 1.2 (or ≤ -1.2); and of these, 1182 DE genes were expressed higher in abdominal fat of LL chickens, whereas only 505 DE genes were expressed higher in FL chickens (S4 Table). A working list of 607 functional genes, associated with lipid metabolism (lipogenesis, lipolysis, lipid transport, etc.), was compiled from the RNA-Seq datasets using Ingenuity Pathway Analysis software (S5 Table). The Venn diagram (Fig 1) shows the intersection among HE genes, DE genes, and lipid metabolism genes, with 25 genes in common across all three gene sets. A total of 164 DE genes were shared between HE and DE gene lists. And, 108 DE genes are shared between the 1687 DE genes and the 607 lipid metabolism genes. Further, 87 genes are shared between the 900 HE genes and the 607 lipid metabolism genes.

### Ingenuity Pathway Analysis (IPA) of gene expression

#### Analysis of highest expressed genes in abdominal fat of FL and LL chickens

The 900 highest-expressed (HE) genes in abdominal fat (S3 Table) were submitted to the Ingenuity Knowledge Base (http://www.ingenuity.com/) for functional annotation, mapping of genes to canonical pathways, and identifying gene interaction networks. There were 828 DE genes identified by IPA as “Analysis ready” (i.e., annotated in the Ingenuity Knowledge Base). A summary of the over-represented IPA functional categories found for 828 “Analysis ready” HE genes is presented in Table 2. The major IPA categories represented by the HE genes include the “Top Canonical Pathways, Diseases and Disorders, Molecular and Cellular Functions, Physiological System Development and Function, Top Gene Interaction Networks, and Top Tox [Toxicology] Lists”. Several subcategories under the IPA category “Diseases and Bio Functions” reveal the most prevalent biological processes found in abdominal fat. For example, several subcategories are related to the regulation of adiposity [“Metabolic Disease” (193 genes), “Endocrine System Disorders” (50 genes), “mTOR Signaling” (31 genes), “Insulin Receptor Signaling” (12 genes), and [“Type 2 Diabetes Signaling” (10 genes)]. Additional subcategories of interest are those involved in metabolism [“Lipid Metabolism” (164 genes), “Carbohydrate Metabolism” (65 genes), “Glycolysis I” (8 genes)] and hemostasis [“Thrombin Signaling” (23 genes)].

**Table 2:** Ingenuity Pathway Analysis of highest-expressed (HE) genes in abdominal fat of FL and LL.

**Top Canonical Pathways**	**p-value**	**Overlap**	**Ratio**
EIF2 Signaling	1.60E-27	27.00%	50/185
Integrin Signaling	1.00E-18	20.90%	42/201
Epithelial Adherens Junction Signaling	8.09E-18	24.00%	35/146
ILK Signaling	3.91E-15	19.40%	36/186
Caveolar-mediated Endocytosis Signaling	3.58E-13	29.60%	21/171
**Diseases and Disorders**	**p-value**	**# Genes**	
Cancer	1.21E-08–1.20E-28	747	
Organismal Injury and Abnormalities	1.21E-08–1.20E-28	754	
Infectious Disease	1.08E-08–1.20E-27	196	
Developmental Disorder	4.85E-09–1.86E-13	160	
Hereditary Disorder	9.81E-09–373.E-26	107	
**Molecular and Cellular Functions**	**p-value**	**# Genes**	
Cellular Growth and Proliferation	1.22E-08–2.9.E-51	420	
Cellular Movement	1.21E-08–4.98E-48	302	
Cell Death and Survival	1.28E-08–5.35E-37	372	
Cellular Development	1.15E-05–1.67E-31	385	
Cellular Assembly and Organization	5.40E-09–2.95E-30	271	
**Physiological System Development and Function**	**p-value**	**# Genes**	
Cardiovascular System	1.25E-08–7.38E-36	208	
Organismal Development	7.47E-09–9.24E-34	342	
Organismal Survival	3.50E-10–5.38E-32	282	
Tissue Development	8.09E-09–5.35E-27	313	
Immune Cell Trafficking	6.36E-09–5.58E-20	120	
**Top Gene Interaction Networks**		**Score**	
Cancer, Organismal Injury and Abnormalities, Respiratory Disease		45	
Metabolic Disease, Neurological Disease, Psychological Disorders		42	
Cell Morphology, RNA Post-Transcriptional Modification, Connective Tissue		42	
Nucleic Acid Metabolism, Small Molecule Biochemistry		42	
Embryonic Development, Organismal Development, Tissue Development		40	
**Top Toxicology List**	**p-value**	**Overlap**	**Ratio**
Cardiac Hypertrophy	1.11E-11	12.20%	48/395
Hepatic Fibrosis	3.61E-10	12.20%	21/99
Renal Necrosis/Cell Death	9.46E-10	10.30%	1/494
PPAR/RXR Activation	1.38E-09	15.30%	28/183
Mechanism of Gene Regulation by PPARs	4.22E-08	18.90%	18/95

A total of the 900 highest-expressed (HE) genes from RNA-Seq analysis were submitted to IPA, which provided 828 “Analysis Ready” HE genes for functional annotation and mapping to canonical pathways and gene interaction networks. *P*-values were determined by IPA software using Fisher’s Exact Test as described by Ingenuity. The percent overlap and ratio were calculated from the number of observed genes compared to the number of known genes for that category in the Ingenuity Knowledge Base.

Several categories under the IPA Top Tox Lists (Table 2) are also of particular interest. Of the 21 HE genes associated with “Hepatic Fibrosis”, 17 HE genes are expressed higher in abdominal fat of the LL chickens, including 5 collagen genes (*COL3A-COL6A*), fibronectin 1 (*FN1*), spondin 1 and 2 (*SPON1*, *SPON2*), fibrillin 1 (*FBN1*), transforming growth factor, beta receptor II (*TGFBR2*) and thrombospondin 1 (*THBS1*). The “PPAR/RXR Activation” category includes 28 genes that are highly expressed in the FL and involved in lipid synthesis [thyroid hormone responsive spot 14 alpha (*THRSPA*), stearoyl-CoA desaturase (*SCD1*), fatty acid synthase (*FASN*), sterol regulatory element binding factor 2 (*SREBP2*), lipoprotein lipase (*LPL*) and glycerol-3-phosphate dehydrogenase 1 (*GDP1*)]. Likewise, the “Mechanism of Gene Regulation by PPARs” category includes 18 HE genes.

**Fig 2:**
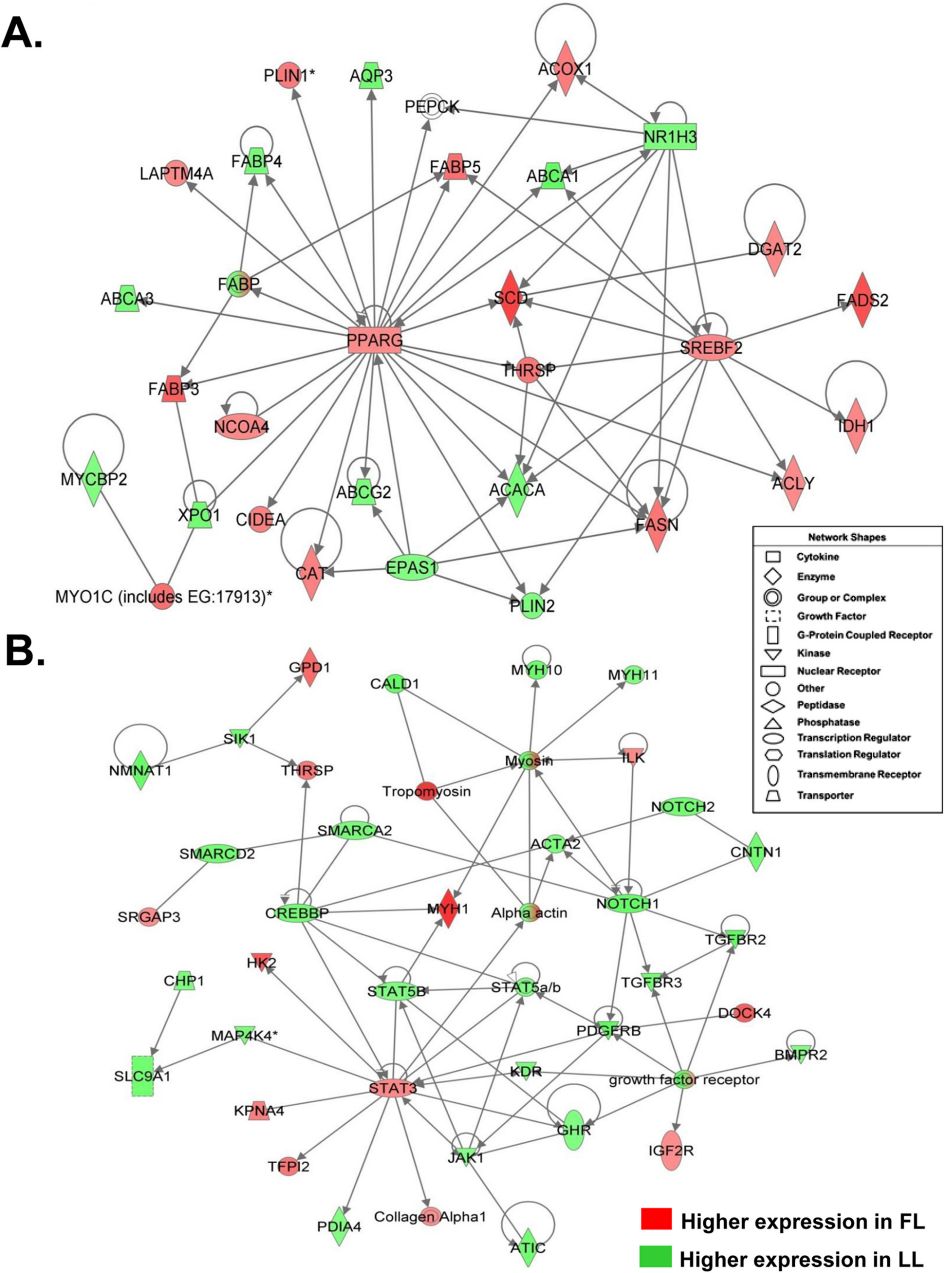
Gene interaction networks of highest-expressed (HE) genes in abdominal fat of chickens associated with lipogenesis and adipogenesis. Functional gene interaction networks were identified by Ingenuity Pathway Analysis (IPA). Genes are colored based on fold-change values determined by RNA-Seq analysis, where the red-color symbols signify higher expression in FL chickens and green-color gene symbols indicate higher expression in LL chickens. The false discovery rate (FDR) and fold-difference cutoff were not used in this functional analysis of the highest-expressed (HE) genes, which is simply based on a high number of reads mapped to known transcripts. Each gene was assigned a shape and function by IPA as shown in the “Network Shapes” legend inset. The direct gene interaction network in the top panel (**A**) was functionally annotated by IPA as “Lipid Metabolism, Molecular Transport, and Small Molecular Biochemistry”, which emphasizes transcriptional regulation of adipogenesis and lipogenesis. The direct gene interaction network in panel (**B)** was functionally annotated by IPA as related to “Cellular Development, Cellular Growth and Proliferation, and Cellular Movement”.

Many HE genes found in abdominal fat of the FL and LL chickens are key transcriptional regulators of lipogenesis and adipogenesis (Fig 2). For example, several transcription factors [*SREBF2*, *THRSP*, nuclear receptor subfamily 1, group H, member 3 (*NR1H3*) or liver-activated receptor alpha (*LXRA*), and peroxisome proliferator-activated receptor gamma (*PPARG*)] interact with each other and ultimately effect the transcription of several downstream target genes (Fig 2A). Some HE targets of *SREBF2* include fatty acid desaturase 2 (*FADS2*), acetyl-CoA carboxylase alpha (*ACACA*), *SCD*, *FASN*, ATP citrate lyase (*ACLY*), perilipin 2 (*PLIN2*), isocitrate dehydrogenase 1 (*IDH1*) and *THRSP*. The transcription factor *THRSP*, which itself targets *ACACA*, *FASN* and *SCD*, is also regulated by *PPARG*. Further, *PPARG* has numerous HE target genes that regulate lipid metabolism [acyl-Coenzyme A oxidase 1, palmitoyl (*ACOX1*), fatty acid binding protein 3–5 (*FABP3*, *FABP4* and *FABP5*), catalase (*CAT*), perilipin 1 (*PLIN1*), lysosomal-associated protein transmembrane 4 A (*LAPTM4A*) and nuclear receptor subfamily 1, group H, member 3 (*NR1H3* or *LXRA*)].

The target genes that are regulated by these transcription factors (Fig 2A) belong to a group of genes functionally associated with “Lipid Metabolism”. Some genes associated with “Lipid Metabolism” are more specifically involved in: fatty acid synthesis, elongation, and desaturation [*ACACA*, *ACOX1*, acyl-CoA dehydrogenase, long chain (*ACADL*), acyl-CoA synthetase long-chain family member 1 (*ACSL1*) and diacylglycerol O-acyltransferase 2 (*DGAT2*), fatty acid elongase 1 (*ELOVL1*), *FASN*, *FADS2*, and *SCD*], fatty acid transport [fatty acid binding protein 4 (*FABP3*, *FABP4* and *FABP5*)], and adipokine signaling [adiponectin (*ADIPOQ*) and *LPL*]. Some other HE genes that are involved in lipid metabolism include diacylglycerol kinase, zeta (*DGKZ*), insulin-like growth factor 2 receptor (*IGF2R*), insulin-like growth factor binding protein 2 and 7 (*IGFBP2* and *IGFBP7*, respectively), malate dehydrogenase 2, NAD (*MDH2*), and somatostatin receptor 2 (*SSTR2*).

Signal transducers and activators of transcription 3 and 5B (*STAT3* and *STAT5B*) are also highly expressed in adipose tissue of FL and LL chickens, respectively (Fig 2B). *STAT3* is a binding partner of *STAT5B*, *GHR*, and mitogen-activated protein kinase kinase kinase kinase 4 (*MAP4K4*) and directly targets hexokinase 2 (*HK2*), tissue factor pathway inhibitor 2 (*TFPI2*) and protein disulfide isomerase family A, member 4 (*PDIA4*). Conversely, several genes that target *STAT3* are also highly expressed [Janus kinase 1 (*JAK1*), kinase insert domain receptor (*KDR*), platelet-derived growth factor receptor, beta polypeptide (*PDGFRB*), and CREB binding protein (*CREBBP*), which also targets *THRSP*]. Another gene up-regulated in abdominal fat of FL chickens at 7 wk was the glycolytic enzyme glycerol-3-phosphate dehydrogenase 1 (*GPD1*), an important regulator of adiposity. Furthermore, NOTCH cellular signaling and growth regulation were significant processes found in abdominal fat of FL and LL chickens. Notch 1 (*NOTCH1*) has interactions with *PDGFRB*, transforming growth factor, beta receptor II and III (*TGFBR2* and *TGFBR3*), actin, alpha 2 (*ACTA2*), contactin 2 (*CNTN2*) and SWI/SNF related, matrix associated, actin dependent regulator of chromatin, subfamily a, member 2 (*SMARCA2*).

#### Analysis of differential gene expression between FL and LL chickens

The dataset of 1687 DE genes (S4 Table) was submitted to Ingenuity Pathway Analysis (IPA) (http://www.ingenuity.com/) for functional annotation and mapping to canonical metabolic and regulatory pathways. From these, a total of 1322 DE genes were identified as “Analysis ready” by IPA. A summary of the Ingenuity Pathway Analysis of these DE genes is presented in Table 3, which includes the major functional categories: “Top Canonical Pathways, Diseases and Disorders, Molecular and Cellular Functions, Physiological System Development and Function, Top Gene Interaction Networks, Top Tox [Toxicology] Lists, and Top 10 Up-regulated and Down-regulated Genes”. Annotated lists are provided for DE genes assigned by IPA to the “Top Canonical Pathways” (S6 Table) and “Top Tox Lists” (S7 Table) functional categories. A total of 1687 differentially-expressed (DE) genes from RNA-Seq analysis were submitted to IPA, which provided 1322 “Analysis Ready” DE genes for functional annotation and mapping to canonical pathways and gene interaction networks. P-values were determined by IPA software using Fisher’s Exact Test as described by Ingenuity. The percent overlap and ratio were calculated for the number of observed genes compared to the number of known genes in the Ingenuity Knowledge Base for that category. Adipose genes with positive expression ratios are expressed at higher levels in FL cockerels, whereas genes with negative expression ratios are more abundant in abdominal fat of the LL.

**Table 3:** Ingenuity Pathway Analysis of DE Genes in Abdominal Fat of FL and LL Cockerels (7 wk).

**Top Canonical Pathways**		**p-value**		**Overlap**	**Ratio**
Adipogenesis pathway		4.13E-06		18.10%	23/127
Glucocorticoid Receptor Signaling		2.61E-05		13.10%	36/275
Axonal Guidance Signaling		5.33E-05		13.10%	49/433
Hepatic Fibrosis/Hepatic Stellate Cell Activation		1.29E-04		13.60%	27/198
RAR Activation		1.61E-04		13.70%	26/190
**Molecular and Cellular Functions**		**p-value**			**#DE Genes**
Cellular Growth and Proliferation		1.66E-04–1.48E-19			489
Cellular Movement		1.62E-04–5.59E-18			316
Cellular Development		1.66E-04–7.72E-17			451
Cell Morphology		1.46E-04–4.76E-14			350
Cellular Assembly, Organization		6.37E-05–1.86E-13			260
**Physiological System Development and Function**		**p-value**			**# DE Genes**
Cardiovascular System		1.49E-04–3.28E-21			238
Organismal Survival		1.22E-04–2.28E-18			340
Organismal Development		1.58E-04–1.50E-17			443
Embryonic Development		1.37E-04–8.86E-15			303
Renal and Urological System		5.99E-05–3.37E-13			77
**Top Gene Interaction Networks**			**Score**		
Cancer, Organismal Injury and Abnormalities, Hematological Disease			43		
Cell Death and Survival, Gene Expression, Cell Cycle			43		
Lipid Metabolism, Small Molecule Biochemistry, Molecular Transport			39		
Cell Signaling, Post-Translational Modification, Embryonic Development			39		
Cardiovascular Disease, Carbohydrate Metabolism, Molecular Transport			36		
**Top Tox List**		**p-value**		**Overlap**	**Ratio**
Cardiac Hypertrophy		1.01E-06		12.30%	28/227
Liver Proliferation		5.27E-04		12.30%	28/227
Oxidative Stress		7.58E-04		19.30%	11_57
TGF-Signaling		1.47E-03		15.60%	14/90
**Top Up-regulated Genes**	**FL/LL ratio**	**Top Down-regulated Genes**			**FL/LL ratio**
*HS3ST5*	8.024	*KRT7*			-3.685
*CLSTN2*	3.724	*MARCO*			-3.644
*LAMB3*	3.488	*SLC7A7*			-3.446
*GJB1*	2.866	*GIF*			-2.859
*XK*	2.841	*TMEM237*			-2.741
*GREM1*	2.806	*AQP1*			-2.561
*SMOC1*	2.601	*C7*			-2.551
*ST14*	2.551	*GUCY1B2*			-2.527
*NTN1*	2.543	*KLF5*			-2.488
*SOST*	2.543	*ALDH1A1*			-2.444

A total of 1687 differentially-expressed (DE) genes from RNA-Seq analysis were submitted to IPA, which provided 1322 “Analysis Ready” DE genes for functional annotation and mapping to canonical pathways and gene interaction networks. P-values were determined by IPA software using Fisher’s Exact Test as described by Ingenuity. The percent overlap and ratio were calculated for the number of observed genes compared to the number of known genes in the Ingenuity Knowledge Base for that category. Adipose genes with positive expression ratios are expressed at higher levels in FL cockerels, whereas genes with negative expression ratios are more abundant in abdominal fat of the LL.

The top canonical pathway populated by 23 DE genes found in abdominal fat of the FL and LL chickens at 7 wk was the “Adipogenesis Pathway” (Table 3). Among the 8 DE genes up-regulated in the FL were frizzled class receptor 6 (*FZD6*), fibroblast growth factor receptor 3 (*FGFR3*), lipin 1 (*LPIN1*), CCAAT/enhancer binding protein (C/EBP) alpha (*CEBPA*), and perilipin 1 (*PLIN1*) (S7 Table), A total of 15 DE genes were expressed higher in the LL chickens; and among these, 12 genes encode transcription factors including Kruppel-like factor 5 (*KLF5*), nuclear receptor subfamily 2, group F, member 2 (*NR2F2* or *COUPTFII*), early B-cell factor 1 (*EBF1*), SMAD family member 5 (*SMAD5*) and hypoxia inducible factor 1, alpha (*HIF1A*), The “Glucocorticoid Receptor (GR) Signaling” pathway contained 36 DE genes (10 genes up-regulated in the FL; 26 genes up-regulated in the LL). The highly-expressed DE genes in the FL included annexin A1 (*ANXA1*), plasminogen activator, urokinase (*PLAU*), *CEBPA*, Harvey rat sarcoma viral oncogene (*HRAS*), two RNA polymerases (*TAF13* and *POLR2H*), and two heat-shock proteins (*HSPA2* and *HSPA8*). A total of 14 transcription regulators related to GR signaling were more abundant in visceral fat of the LL, including progesterone receptor (*PGR*), androgen receptor (*AR*), nuclear receptor coactivator 2 and 3 (*NCOA2; NCOA3*), nuclear factor of activated T cells, cytoplasmic, calcineurin dependent 2 and 3 (*NFATC2*; *NFATC3)*, and E1A-binding protein p300 (*EP300*). Of the 49 DE genes assigned to the “Axonal Guidance Signaling” pathway, 16 genes were up-regulated in the FL and 33 genes were more abundant in the LL, including 6 peptidases and 13 kinases. The “Hepatic Fibrosis” pathway also shows over-representation of 22 DE genes in visceral fat of the LL that encode 8 types of collagen, myosin, heavy chain (*MYH10*), 8 growth factor receptors [fibroblast growth factor receptor 2 (*FGFR2*), platelet-derived growth factor receptor, alpha and beta polypeptides (*PDGFRA*; *PDGFRB*), interleukin 1 receptor, type I (*IL1R1*), insulin-like growth factor 1 receptor (*IGF1R*), interferon gamma receptor 1 (*IFNGR1*), transforming growth factor, beta receptor II (*TGFBR2*), and angiotensin II receptor, type 1 (*AGTR1*)], and 4 growth factors (ligands) [(transforming growth factor, beta 2 (*TGFB2*), *PDGFRB*, tumor necrosis factor (ligand) superfamily, member 10 (*TNFSF10*) and c-fos induced growth factor (*FIGF*)]. In contrast, the FL chickens showed up-regulation of only five genes associated with fibrosis, namely three receptors (tumor necrosis factor receptor superfamily, member 1B (*TNFRSF1B*), endothelin receptor type B (*EDNRB*) and interleukin 1 receptor accessory protein-like 2 (*IL1RAPL2*), *SMAD7* and platelet derived growth factor C (*PDGFC*)]. The “RXR Activation” pathway was similarly over-represented with highly-expressed DE genes from the LL (23 genes, including retinoic acid receptor beta (*RARB*), *EP300*, and Janus kinase 2 (*JAK2*), whereas only retinoid X receptor gamma (*RXRG*), aldehyde dehydrogenase family 1, subfamily A2 (*ALDH1A2*) and *SMAD7* were expressed higher in the FL birds.

The “Top Tox” function identified by IPA for DE genes was “Cardiac Hypertrophy” (Table 3; S7 Table), which list 13 genes that are up-regulated in FL birds [i.e., *PLAU*, thioredoxin (*TXN*), *FABP3*, Parkinson protein 7 (*PARK7*), galactosidase, alpha (*GLA*), etc.] and 35 genes expressed higher in LL abdominal fat [i.e, *KLF5*, *FN1*, *AGTR1/2*, periostin (*POSTN*), angiopoietin 1 (*ANGPT1*), and]. The “Liver Proliferation” category includes 11 DE genes up-regulated in the FL and 17 DE genes expressed higher in the LL. Among the DE genes highly expressed in FL adipose tissue were leukotriene B4 receptor (*LTB4R*), protein S alpha (*PROS1*), chemokine (C-X-C motif) receptor 4 (*CXCR4*), follistatin (*FST*), and *CEBPA*. The DE genes over-expressed in the LL include midkine or neurite growth-promoting factor 2 (*MDK*), pleiotrophin (*PTN*), bone morphogenetic protein 7 (*BMP7*), cannabinoid receptor 1 (*CNR1*), *AGTR1*, and *IGF1R* as examples. The “Oxidative Stress” response shows 8 genes expressed higher in FL and 3 up-regulated in the LL. The “TGF-β signaling” function includes 3 genes up-regulated in the FL, whereas 11 DE genes were more abundant in the LL adipose tissue. Other interesting canonical pathways identified by IPA were “Renin-Angiotensin Signaling” (13 DE genes/109 known genes) “TR/RXR Activation” (11/85), “NF-κB Signaling” (22/173), and “Coagulation System” (8/35). Differential regulation of the IPA “Molecular and Cellular Function” subcategory “Lipid Metabolism” (173 DE genes) is of particular interest to this study.

**Fig 3:**
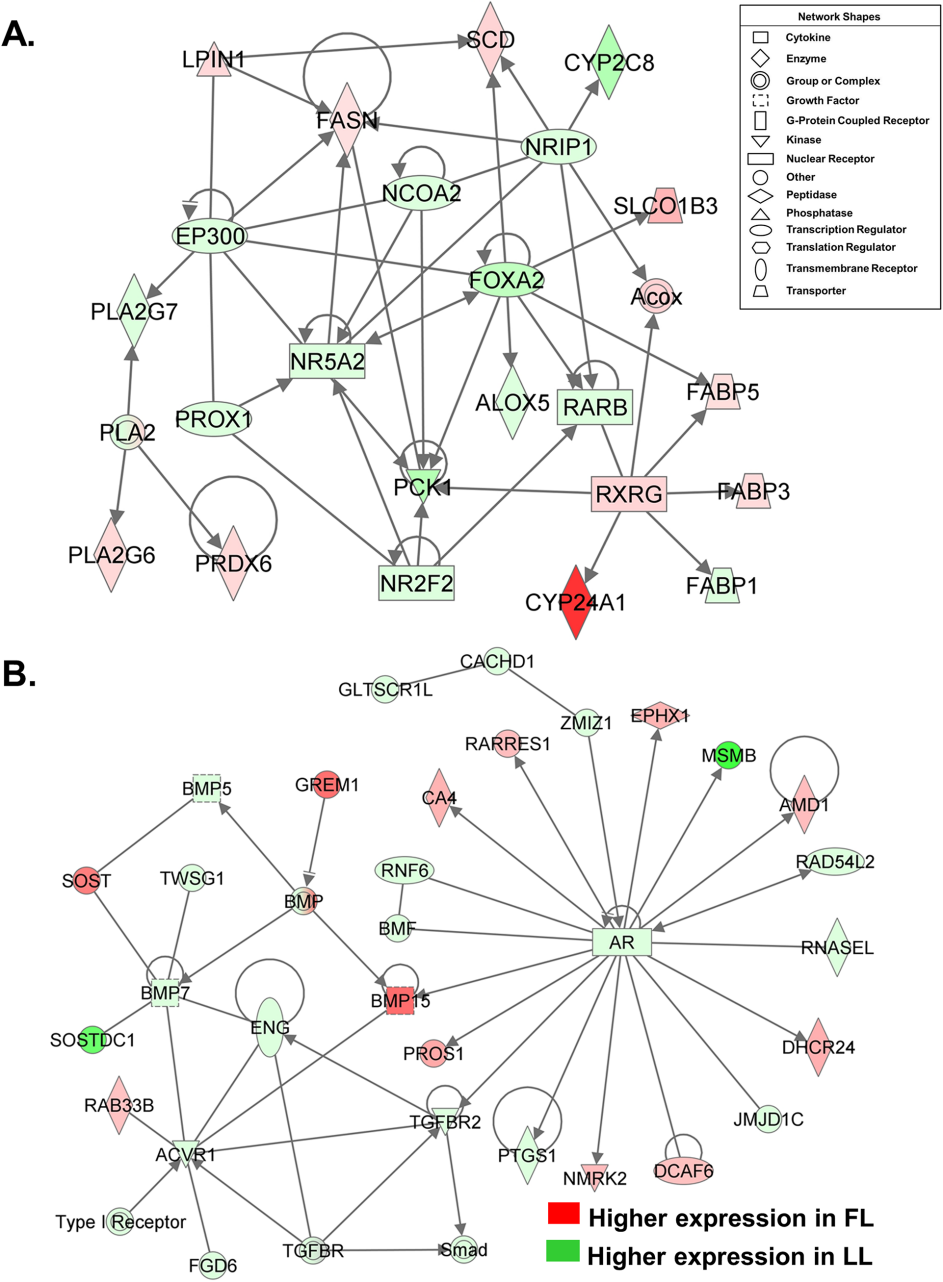
Gene interaction networks in abdominal fat of FL and LL chickens at 7 wk. Functional gene networks were identified by IPA, DE genes are colored based on fold-change values (FL/LL) from RNA-Seq analysis. Each gene was assigned a shape by IPA according to its function as shown in the “Network Shapes” box. The top panel (**A**) shows a direct gene network annotated by IPA as “Lipid Metabolism, Small Molecule Biochemistry and Molecular Transport” that involves the interaction of several transcription factors, ligand-activated nuclear receptors, lipogenic enzymes and fatty acid transporters. The bottom direct gene interaction network (**B**) is centered on the androgen receptor (AR) and several direct targets of the AR including transforming growth factor, beta receptor II (*TGFBR2* **)** and bone morphogenetic protein 15 (*BMP15*).

A large group of DE genes involved in lipid metabolism were identified by IPA (Fig 3A). Eleven lipogenic genes [lipin 1 (*LPIN1*), *FASN*, *SCD*, *RXRG*, *FABP3*, *FABP5*, cytochrome P450, family 24, subfamily A, polypeptide 1 (*CYP24A1*), phospholipase A2, group VI (cytosolic, calcium-independent) (*PLA2G6*), peroxiredoxin 6 (*PRDX6*), solute carrier organic anion transporter family, member 1B3 (*SLCO1B3*) and acyl-CoA oxidase 2 (*ACOX2*)] were up-regulated in abdominal fat of FL chickens. Five transcription factors [*EP300*, prospero homeobox 1 (*PROX1*), *NCOA2*, forkhead box A2 (*FOXA2*), and nuclear receptor interacting protein 1 (*NRIP1*)], three ligand-activated nuclear receptors [liver receptor homolog–1 (*NR5A2*), chicken ovalbumin upstream promoter transcription factor II (*COUPTFII* or *NR2F2*), and *RARB*], three metabolic enzymes [arachidonate 5-lipoxygenase (*ALOX5*), cytochrome P450, family 2, subfamily C, polypeptide 8 (*CYP2C8*) and phospholipase A2, group VII (*PLA2G7*)], phosphoenolpyruvate carboxykinase 1, cytosolic (*PCK1*), which is the rate-limiting enzyme in gluconeogenesis, and liver fatty acid binding protein 1 (*FABP1*) were up-regulated in visceral fat of LL chickens. Another gene interaction network (Fig 3B) centered on the androgen receptor (*AR*) was expressed higher in the LL, which shows numerous direct targets of the AR, including bone morphogenetic protein 15 (*BMP15*, up-regulated in the FL) and *TGFBR2* (up-regulated in the LL), which interact with TGF family members. Three additional members of the TGFBR family are of particular interest: gremlin 1, DAN family BMP antagonist (*GREM1*, up-regulated in the FL), endoglin (*ENG*), and activin A receptor, type I (*AVR1*), which were up-regulated in visceral fat of the LL. Other direct targets of AR that were up-regulated in abdominal fat of the FL chickens include carbonic anhydrase IV (*CA4*), retinoic acid receptor responder 1 (*RARRES1*), epoxide hydrolase 1 (*EPHX1*), 24-dehydrocholesterol reductase (*DHCR24*) and protein S-alpha (*PROS1*).

**Fig 4:**
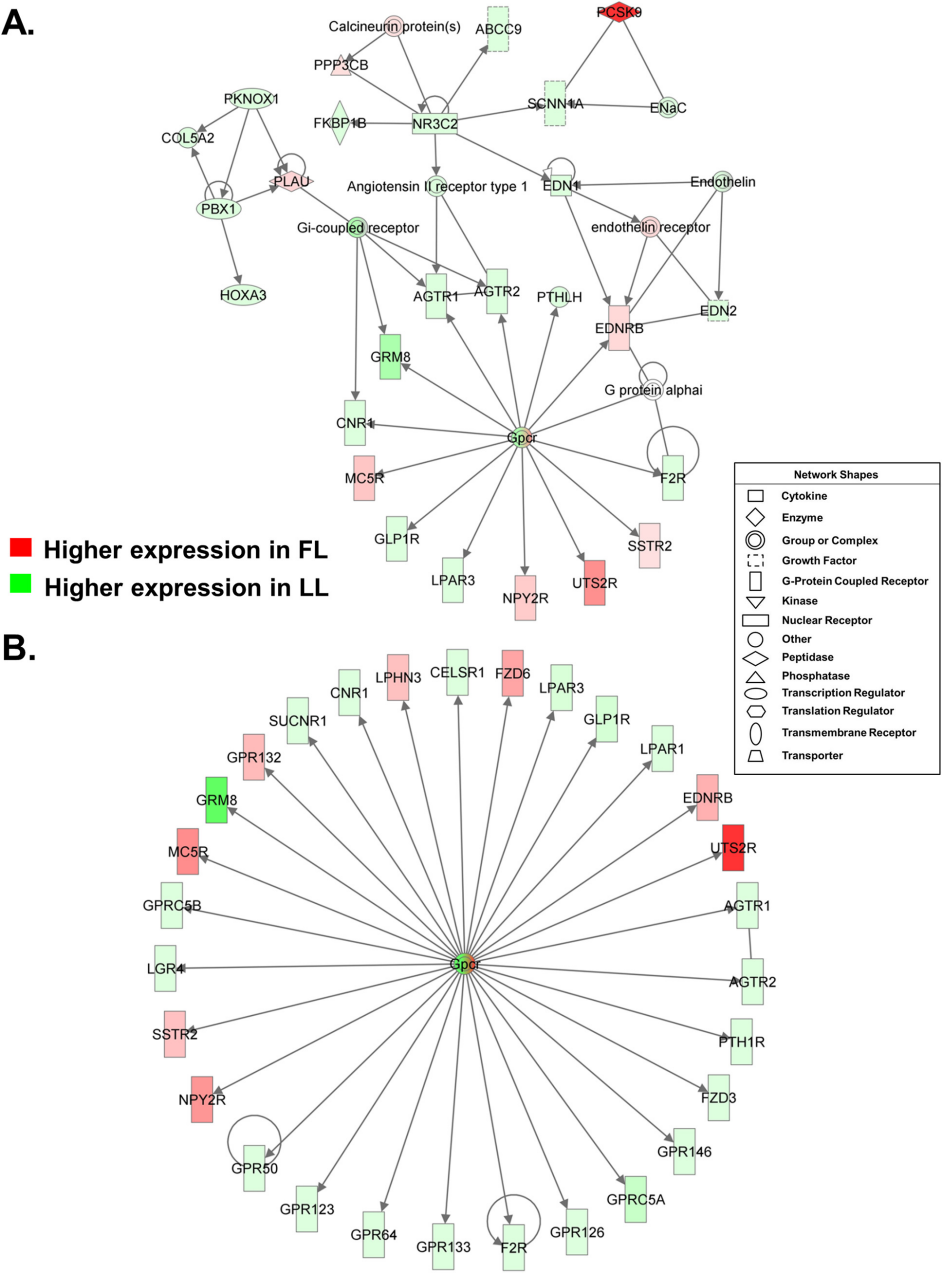
Differential expression of G-protein coupled receptors in abdominal fat of FL and LL chickens. (**A**) Gene interaction network of G-protein coupled receptors (GPCR), some of which interact with regulators of angiogenesis and blood pressure. Visceral adipose tissue shows an extensive over-representation of 29 GPCRs (**B**), where 21 DE genes are over-expressed in the LL and 8 DE genes are expressed higher in FL chickens. Many of these GPCRs could be considered as “ectopically expressed” in adipose tissue.

Numerous G-protein coupled receptors (GPCR) were differentially expressed in abdominal fat of FL and LL chickens at 7 wk (Fig 4). Interestingly, three neuropeptides receptors [melanocortin (*MC5R*), somatostatin (*SSTR2*) and neuropeptide Y, (*NPY2R*)] were up-regulated in visceral fat of the FL chickens (Fig 4A). The urotensin 2 receptor (*UTS2R*) and endothelin receptor B (*EDNRB*) were also higher in FL chickens, while two ligands for the endothelin receptor (endothelin 1 and 2; *EDN1* and *EDN2*) were expressed higher in the LL. Other genes up-regulated in LL chickens were the G-coupled protein receptors for glucagon-like peptide 1 (*GLP1R*), lysophosphatidic acid (*LPAR3*), glutamate (*GRM8*), cannabinoid (*CNR1*), thrombin (*F2R*), and angiotensin II [angiotensin II receptor, type 1 and 2 (*AGTR1* and *AGTR2*)]. The mineralocorticoid nuclear receptor [MCR or nuclear receptor subfamily 3, group C, member 2 (*NR3C2*)], several of its targets [sodium channel, non-voltage-gated 1 alpha subunit (*SCNN1A*), FK506 binding protein 1B (*FKBP1B*), ATP-binding cassette, sub-family C (CFTR/MRP), member 9 (*ABCC9*), *AGTR1* and *EDN1*)], parathyroid hormone-like hormone (*PTHLH*) and homeobox A3 (*HOXA3*) were also higher in abdominal fat of the LL chickens. Plasminogen activator, urokinase (*PLAU*) is responsible for the conversion of plasminogen to plasmin (an important step in the fibrinolytic pathway) was up-regulated in FL chickens. The most abundant gene found in this interaction network of FL abdominal fat was proprotein convertase subtilisin kexin type 9 (*PCSK9*), a potent inhibitor of the low-density lipoprotein receptor (LDLR) and regulator of plasma LDL cholesterol levels.

RNA-Seq analysis revealed 29 GCPRs that were differentially expressed in abdominal fat of the FL and LL chickens at 7 wk of age (Fig 4B). Eight GCPR genes (*EDNRB*, *FZD6*, G protein-coupled receptor 132 (*GPR132*), latrophilin 3 (*LPHN3*), *MC5R*, neuropeptide Y receptor 2 (*NPYR2*), *SSTR2* and *UTS2R*) were expressed at higher levels in the FL birds; whereas, 21 GCPRs were over-expressed in abdominal fat of the LL. Many of these differentially expressed GCPRs are usually expressed in tissues other than abdominal fat and have a variety of functions including cell adhesion and signaling, metabolism, angiogenesis, vasomotor tone, cell differentiation, embryonic development, etc.

**Fig 5:**
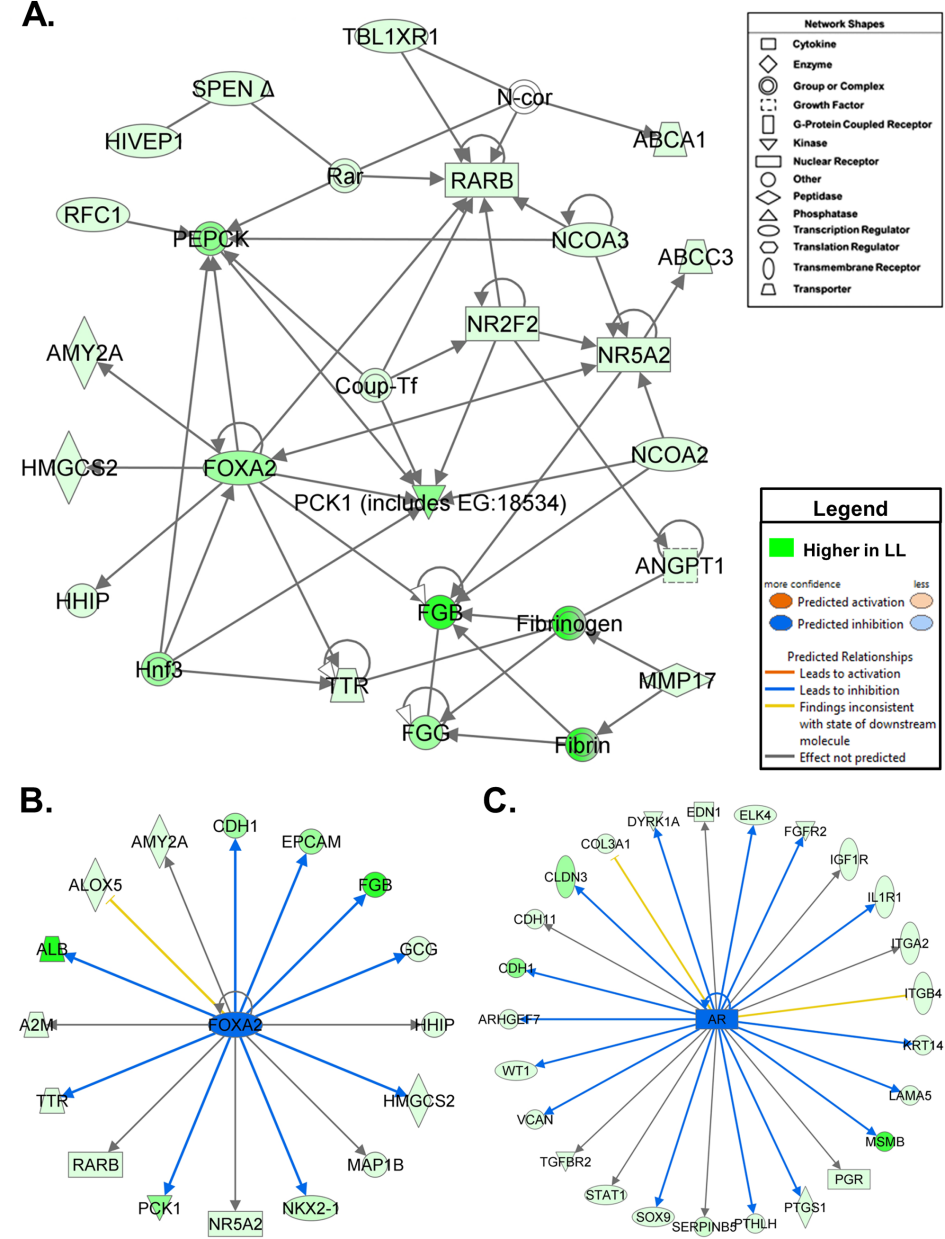
Gene interaction network of up-regulated genes and direct targets of up-regulated transcription factors in abdominal fat of the LL chickens. (**A**) This direct gene interaction network of up-regulated DE genes in the LL chickens was identified by IPA as “Developmental Disorder, Hematological Disease, or Hereditary Disorder”. (**B**) The Ingenuity Upstream Regulator Analysis predicts inhibition of forkhead box A2 (FOXA2; blue-colored symbol with a z-score of -2.52), since 9 of its 17 direct targets have down-regulated expression ratios (FL/LL), which is consistent with inhibition of *FOXA2* (i.e., down-regulated in FL or up-regulated in LL). (**C**) Likewise, the predicted inhibition of the androgen receptor (*AR*; blue-colored symbol with a z-score of -3.04) would lead to inhibition (blue arrows) of its direct targets, since 15 of 27 genes are inhibited in the FL (i.e., a reduced FL/LL ratio or up-regulated in the LL). According to RNA-Seq analysis, the expression of both *FOXA2* (fold-change of -10.7) and *AR* (fold-change of -1.4) was higher in the LL.

Examination of DE genes over-expressed in abdominal fat of the LL chickens (Fig 5A) revealed an interaction network of several transcriptional regulators [(*FOXA2*, *NCOA2*, *NCOA3*, transducin (beta)-like 1 X-linked receptor 1 (*TBL1XR1*), spen family transcriptional repressor (*SPEN*) and human immunodeficiency virus type I enhancer binding protein 1 (*HIVEP1*)], transporters [transthyretin (*TTR*) and ATP-binding cassette, sub-family C, member 3 (*ABCC3*) ATP-binding cassette, sub-family A, member 1 (*ABCA1*)], enzymes [phosphoenolpyruvate carboxykinase 1 (*PCK1*), 3-hydroxy-3-methylglutaryl-CoA synthase 2 (*HMGCS2*) and amylase, alpha 2A (*AMY2A*)] and ligand-activated nuclear receptors [*NR2F2*, nuclear receptor subfamily 5, group A, member 2 (*NR5A2*) and *RXRB*]. Additional direct DE gene targets of the up-regulated transcription factors *FOXA2* (Fig 5B) and *AR* (Fig 5C) in the LL were identified by the Ingenuity Upstream Regulatory Analysis, which predicts that these two upstream regulators are inhibited (blue-colored symbols and edges), since their direct target genes are also down-regulated in the FL (i.e., expressed higher in the LL). This network of up-regulated genes, which interact with six transcription factors and numerous up-regulated direct targets of *FOXA2* and *AR* in abdominal fat of the LL (Fig 5) clearly implicates intense transcriptional regulation of the lean phenotype. In particular, *FOXA2* appears to play a critical role in restricting visceral adipose accretion in the LL, since it directly up-regulates expression of fibrinogen beta (*FGB*, a key blood-clotting protein), glucagon (*GCG*, a lipolytic pancreatic hormone), *PCK1* (the key enzymatic regulator of gluconeogenesis), *HMGCS2* (which catalyzes the first reaction in ketogenesis), two major transport proteins (*ALB* and *TTR*), and alpha-2-macroglobulin (*A2M*, a protease inhibitor and clinical biomarker of type 2 diabetes).

**Fig 6:**
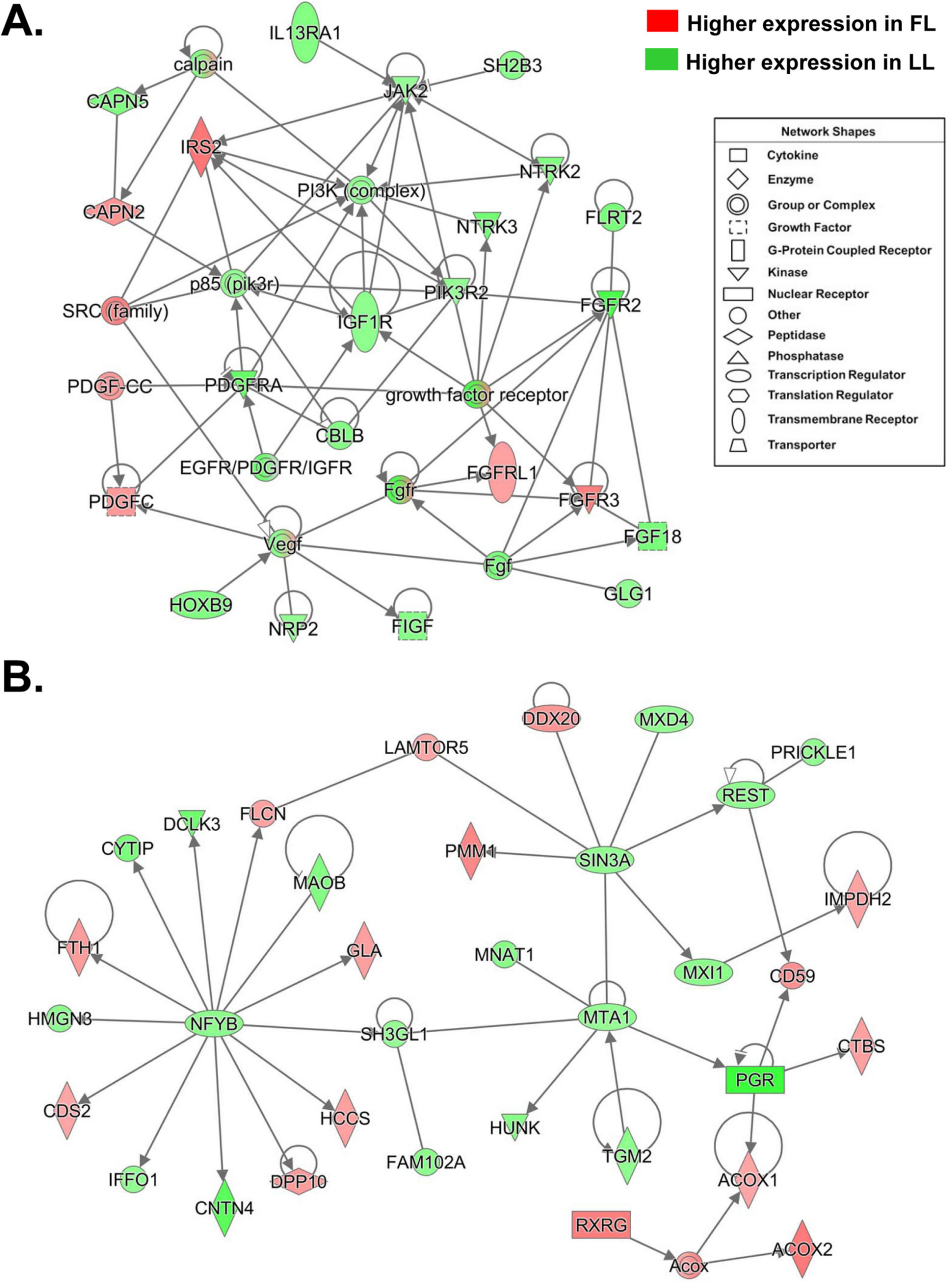
Gene interaction networks involved in growth factor signaling (A) and (B) transcription factor regulation. **(A)** This gene network is highly populated by genes involved in growth factor signaling and functionally annotated by IPA as “Cellular Growth and Proliferation, Cell Cycle and Cancer”. **(B)** This gene interaction network is populated by several transcription factors up-regulated in the LL chickens and functionally annotated by IPA as “Cell Death and Survival, Gene Expression, Cell Cycle”.

Another interesting gene interaction network involved in growth factor signaling was also highly represented by DE genes found in the LL adipose tissue (Fig 6A). Of the 17 genes up-regulated in the LL, there were 7 growth factor receptors (*FGFR2*, *PDGFRA*, fibronectin leucine rich transmembrane protein 2 (*FLRT2*), neurotrophic tyrosine kinase, receptor, type 2 (*NTRK2*), interleukin 13 receptor, alpha 1 (*IL13RA1*), and *IGF1R*), 2 growth factors [fibroblast growth factor 18 (*FGF18*) and *FIGF*], 4 kinases [neurotrophic tyrosine kinase, receptor, type 3 (*NTRK3*), phosphoinositide-3-kinase, regulatory subunit 2 (beta) (*PIK3R2*), neuropilin 2 (*NRP2*), and *JAK2)*], 1 peptidase [calpain 5 (*CAPN5*)], 1 transcription factor [homeobox B9 (*HOXB9*)], and 3 “other” genes [Cbl proto-oncogene B, E3 ubiquitin protein ligase (*CBLB*), SH2B adaptor protein 3 (*SH2B3*) and golgi glycoprotein 1 (*GLG1*)]. In contrast, only five DE genes in this network were up-regulated in the FL, including the insulin receptor substrate 2 (*IRS2*), *FGFR3*, *PDGFC*, fibroblast growth factor receptor-like 1 (*FGFRL1*), and the peptidase (*CAPN2*). The gene interaction network shown in Fig 2B was listed in Table 3, under the IPA “Top Gene Interaction Network” functionally annotated as “Cell Death and Survival, Gene Expression, Cell Cycle”. Fifteen DE genes in this network are up-regulated in the FL; and all appear to be direct targets of multiple transcription factors, which are expressed higher in the LL. Many of the DE genes up-regulated in the FL are enzymes [*ACOX1*, *ACOX2*, CDP-diacylglycerol synthase (phosphatidate cytidylyltransferase) 2 (*CDS2*), IMP (inosine 5'-monophosphate) dehydrogenase 2 (*IMPDH2*), chitobiase, di-N-acetyl- (*CTBS*), holocytochrome c synthase (*HCCS*), galactosidase, alpha (*GLA*), and phosphomannomutase 1 (*PMM1*)], although two highly-expressed DE genes in the FL encode transcription regulators [*RXRG* and DEAD (Asp-Glu-Ala-Asp) box polypeptide 20 (*DDX20*)]. In contrast, 8 of the 19 DE genes that are expressed higher in the LL are transcription factors [(*PGR*, MAX interactor 1, dimerization protein (*MXI1*), nuclear transcription factor Y, beta (*NFYB*), *RE1-*silencing transcription factor (*REST*), Max dimerization protein 4 (*MXD4*), metastasis associated 1 (*MTA1*), prickle homolog 1 (*PRICKLE1*) and SIN3 transcription regulator family member A (*SIN3A*)]. Three enzymes were among the other up-regulated genes in the LL are [transglutaminase 2 (*TGM2*), monoamine oxidase B (*MAOB*), and contactin 4 (*CNTN4*)], while two genes were kinases [(doublecortin-like kinase 3 (*DCLK3*) and hormonally up-regulated Neu-associated kinase (*HUNK*)].

Ingenuity Upstream Regulator Analysis revealed 16 DE transcription regulators and their DE direct target genes from the RNA-Seq analysis of abdominal fat in the FL and LL cockerels at 7 wk (S8 Table). Only four DE transcription factors were up-regulated in the FL [ELL associated factor 2 (*EAF2*), *SMAD7*, *PARK7* and *CEBPA*]. In contrast, 12 DE transcription regulators were expressed at higher levels in abdominal fat of the LL. The four highest expressed transcription factors in the LL were *KLF5*, *PGR*, homeobox A11 (*HOXA11*), and SWI/SNF related, matrix associated, actin dependent regulator of chromatin, subfamily b, member 1 (*SMARCB1*). The transcription factors in the LL with the greatest number of downstream DE target genes were *EP300* (46 DE genes), *AR* (46 DE genes), *NFYB* (28 DE genes), and *PGR* (25 DE genes). This over-representation of DE upstream regulator genes in the LL emphasizes the importance of enhanced transcriptional regulation in expression of the divergent lean phenotype.

A comparison between the DE and HE gene sets has identified numerous candidate genes that control lipid metabolism and expansion of visceral fat (Table 4). Of these candidate genes, the most remarkable include *FABP3*, *FABP5*, *FADS2*, *FASN*, *GPD1*, *HK2*, *HSD17B7*, *IGFBP2*, *MDH2*, *PGRMC1* and *SCD*. These genes could aid in the transport of lipid (*FABP3* and *FABP5*) and the generation of substrate (*HK2*, *GDP1*, and *MDH2*) for lipid synthesis via the major lipogenic enzymes *FASN* and *SCD*, both of which are highly expressed in abdominal fat of FL chickens. These lipogenic genes are controlled by the interaction of multiple up-stream transcription regulators (*THRSP*, *SREBF2*, *STAT5B*, *PPARG*, *NFKBIA*, *RARB* and *PARK7)*. Other DE and/or HE genes involved in angiogenesis and hemostasis include the natriuretic peptide receptor 3 (*NPR3*), *PROS1*, *PDGFRB*, *EDNRB*, *PLAU*, *PROCR*, and *ANG*.

**Table 4:** Functional genes identified by RNA-Seq analysis of abdominal fat in FL and LL cockerels at 7 wk.

**Differentially Expressed and Highest Expressed Lipid Metabolism Genes**			
**Gene Symbol**	**Gene Name**	**Fold Change**	**Average Reads**
***FABP3***	fatty acid binding protein 3, muscle and heart	1.4	10216
***FABP5***	fatty acid binding protein 5 (psoriasis-associated)	1.3	10800
***FADS2***	fatty acid desaturase 2	1.6	6764
***FASN***	fatty acid synthase	1.2	32362
***GPD1***	glycerol-3-phosphate dehydrogenase 1 (soluble)	1.3	16641
***HK2***	hexokinase 2	1.5	5270
***HSD17B7***	hydroxysteroid (17-beta) dehydrogenase 7	1.5	4293
***IGFBP2***	insulin-like growth factor binding protein 2, 36kDa	2.5	5886
***MDH2***	malate dehydrogenase 2, NAD (mitochondrial)	1.2	5214
***PGRMC1***	progesterone receptor membrane component 1	1.4	22381
***SERINC1***	serine incorporator 1	1.2	8013
***SCD***	stearoyl-CoA desaturase (delta-9-desaturase)	1.7	93949
***ABCA1***	ATP-binding cassette, sub-family A (ABC1), member 1	-1.3	6388
***IGFBP7***	insulin-like growth factor binding protein 7	-1.4	10915
***PDGFRB***	platelet-derived growth factor receptor, beta	-1.3	8198
**Differentially Expressed (DE) Genes**			
***ACOX2***	acyl-CoA oxidase 2, branched chain	1.8	370
***ACSBG2***	acyl-CoA synthetase bubblegum family member 2	1.3	2147
***DHCR24***	24-dehydrocholesterol reductase	1.6	3843
***EDNRB***	endothelin receptor type B	1.6	237
***INSIG2***	insulin induced gene 2	1.3	763
***MCAT***	malonyl CoA:ACP acyltransferase (mitochondrial)	1.4	833
***PLAU***	plasminogen activator, urokinase	1.7	694
***PRKAG2***	protein kinase, AMP-activated, gamma 2 subunit	1.4	1286
***ALDH1A1***	aldehyde dehydrogenase 1 family, member A1	-2.5	646
***ALOX5***	arachidonate 5-lipoxygenase	-1.5	449
***BMP5***	bone morphogenetic protein 5	-1.5	162
***BMP7***	bone morphogenetic protein 7	-1.7	154
***DAGLA***	diacylglycerol lipase, alpha	-1.3	627
***FAR1***	fatty acyl CoA reductase 1	-1.8	424
***FAR2***	fatty acyl CoA reductase 2	-1.3	588
***HPGDS***	hematopoietic prostaglandin D synthase	-1.6	152
***IRS4***	insulin receptor substrate 4	-1.2	669
***RARB***	retinoic acid receptor, beta	-1.5	433
***TNFSF10***	tumor necrosis factor (ligand) superfamily, member 10	-1.4	1641
**Highest Expressed (HE) Genes (not DE)**			
***ACACA***	acetyl-CoA carboxylase alpha		6729
***ACACB***	acetyl-CoA carboxylase beta		10524
***ACAD9***	acyl-CoA dehydrogenase family, member 9		6329
***ACADL***	acyl-CoA dehydrogenase, long chain		5508
***ACLY***	ATP citrate lyase		6716
***ACOX1***	acyl-CoA oxidase 1, palmitoyl		22460
***ACSL1***	acyl-CoA synthetase long-chain family member 1		60335
***ACSS2***	acyl-CoA synthetase short-chain family member 2		7764
***ADIPOQ***	adiponectin, C1Q and collagen domain containing		10438
***ANG***	angiogenin, ribonuclease, RNase A family, 5		5741
***ELOVL1***	ELOVL fatty acid elongase 1		6289
***FABP4***	fatty acid binding protein 4, adipocyte		45632
***GHR***	growth hormone receptor		4754
***LPL***	lipoprotein lipase		163988
***NFKBIA***	nuclear factor of kappa inhibitor, alpha		6990
***PLIN1***	perilipin 1		54722
***PLIN2***	perilipin 2		8175
***PPARG***	peroxisome proliferator-activated receptor gamma		6837
***SREBF2***	sterol regulatory element binding transcription factor 2		7325
***STAT5B***	signal transducer and activator of transcription 5B		9306
***THRSPA***	thyroid hormone responsive Spot 14 protein, alpha		42780

Comparison of highest-expressed (HE) and differentially-expressed (DE) genes identified by RNA-Seq analysis in abdominal fat of FL and LL chickens. Adipose genes with positive fold-change (FL/LL) values are expressed higher in the FL, while genes with a negative fold-change are expressed higher in LL chickens. Paired-end sequence reads for each gene were averaged across both genotypes (4 FL and 4 LL). The reads threshold for highest expressed (HE) genes was >4289 reads/gene.

### Verification of RNA-seq analysis by quantitative RT-PCR

Based on biological function, several candidate genes were selected from the RNA-Seq analysis for qRT-PCR verification (Table 5). Of the genes shown, 41 were differentially expressed in the FL/LL by RNA-Seq analysis (FDR-adjusted *P-*value ≤ 0.05). All 41 genes were significantly different (*P*≤ 0.05) between FL and LL birds by qRT-PCR analysis. Neuropeptide Y (*NPY*) was a candidate gene not significantly different between the FL and LL chickens by either RNA-Seq or qRT-PCR analysis. The glucagon receptor (*GCGR*) was not differentially expressed by RNA-Seq; however, the abundance of *GCGR* in abdominal fat was 1.6 fold higher in the LL chickens by qRT-PCR analysis (*P*≤0.05). The short isoform of chicken growth hormone (*scGH*) was 1.5-fold higher *(P*≤0.05) in FL chickens by qRT-PCR analysis, while differential expression of *scGH* was not indicated by RNA-Seq analysis. Albumin (*ALB*; not shown) was highly up-regulated in abdominal fat of LL chickens by both RNA-Seq (38-fold) and qRT-PCR (73-fold) analyses. Furthermore, the fold change values for this gene set, across both RNA-Seq and qRT-PCR analyses, were highly correlated (r = 0.90) and significant (*P*≤0.001) as determined by Pearson correlation analysis. A complete list of the FL and LL expression means and their standard errors (SEM) for all genes in the qRT-PCR analysis is provided in S9 Table.

**Table 5:** Verification of RNA-Seq gene expression in abdominal fat of FL and LL cockerels.

		qRT-PCR		RNA-Seq	
Symbol	Gene Name	Fold change	P-Value	Fold change	P-Value
***SSTR2***	somatostatin receptor 2	2.84	0.002	1.23	0.011
***BMP15***	bone morphogenetic protein 15	2.82	0.030	2.92	0.019
***FASN***	fatty acid synthase	2.75	0.040	1.24	0.003
***SCD***	stearoyl-CoA desaturase (delta-9-desaturase)	2.47	0.040	1.71	0.028
***WNT4***	wingless-type MMTV integration site family 4	2.35	0.010	2.59	0.001
***MC5R***	melanocortin 5 receptor	2.29	0.040	2.28	0.001
***GREM1***	gremlin 1	2.19	0.001	2.85	0.001
***FAAH***	fatty acid amide hydrolase	1.91	0.010	1.31	0.001
***LPIN1***	lipin 1	1.81	0.010	1.52	0.001
***IRS2***	insulin receptor substrate 2	1.77	0.040	1.72	0.039
***FGFR3***	fibroblast growth factor receptor 3	1.72	0.040	1.75	0.001
***NPY2R***	neuropeptide Y receptor Y2	1.68	0.040	2.09	0.032
***DHCR24***	24-dehydrocholesterol reductase	1.64	0.020	1.59	0.001
***ME1***	malic enzyme 1, NADP(+)-dependent, cytosolic	1.61	0.030	1.76	0.001
***HK2***	hexokinase 2	1.57	0.040	1.49	0.005
***HSD17B7***	hydroxysteroid (17-beta) dehydrogenase 7	1.51	0.050	1.55	0.001
***CYP24A1***	cytochrome P450 family 24 subfamily A,1	1.48	0.080	8.24	0.003
***RXRG***	retinoid X receptor, gamma	1.48	0.040	1.67	0.004
***NPY***	neuropeptide Y	1.38	*N*.*S*.	1.51	*N*.*S*.
***FADS2***	fatty acid desaturase 2	1.37	0.050	1.58	0.001
***PGRMC1***	progesterone receptor membrane component 1	1.36	0.010	1.42	0.001
***AGTR1***	angiotensin II receptor, type 1	-1.21	0.050	-1.37	0.005
***GLP1R***	glucagon-like peptide 1 receptor	-1.37	0.040	-3.75	0.001
***CNR1***	cannabinoid receptor 1 (brain)	-1.39	0.050	-1.35	0.014
***SERPING1***	serpin peptidase inhibitor, clade G, member 1	-1.39	0.040	-1.34	0.001
***HIF1A***	hypoxia inducible factor 1, alpha subunit	-1.42	0.040	-1.23	0.013
***NCOA3***	nuclear receptor coactivator 3	-1.51	0.030	-1.21	0.003
***POSTN***	periostin, osteoblast specific factor	-1.53	0.030	-1.44	0.001
***IGF1R***	insulin-like growth factor 1 receptor	-1.57	0.040	-1.27	0.001
***ENG***	endoglin	-1.67	0.040	-1.39	0.023
***AGTR2***	angiotensin II receptor, type 2	-1.74	0.003	-1.61	0.001
***GCG***	glucagon	-1.74	0.020	-1.811	0.032
***JMJD1C***	jumonji domain containing 1C	-1.76	0.030	-1.21	0.001
***EDN2***	endothelin 2	-1.97	0.001	-1.79	0.006
***GRM8***	glutamate receptor, metabotropic 8	-1.97	0.090	-14.23	0.009
***PGR***	progesterone receptor	-2.08	0.010	-2.03	0.003
***EDN1***	endothelin 1	-2.09	0.010	-1.51	0.001
***EP300***	E1A binding protein p300	-2.22	0.040	-1.26	0.001
***AR***	androgen receptor	-2.45	0.050	-1.35	0.001
***PCK1***	phosphoenolpyruvate carboxykinase 1	-3.11	0.020	-12.61	0.001
***FGFR2***	fibroblast growth factor receptor 2	-3.45	0.050	-2.41	0.004
***TTR***	transthyretin	-4.47	0.040	-3.28	0.01
***ALB***	albumin	-73.2	0.030	-38.43	0.037

Comparisons are shown for 41 DE genes from the RNA-Seq analysis and three additional genes that were not differentially expressed by RNA-Seq analysis, but of interest [*NPY*, *GCGR*, and short chicken growth hormone (*scGH*)]. Fold changes were derived from FL/LL expression ratios of 4 birds/genotype at 7 wk. Positive values correspond to up-regulation in FL chickens, while negative ratios indicate higher expression in abdominal fat of LL cockerels. Pearson correlation analysis showed a high correlation (r = 0.90) and significance (*P*≤0.001) between the relative expression ratio obtained by the two independent analytical methods (qRT-PCR and RNA-Seq). No significant difference (*N*.*S*.) was found for abundance of *NPY* transcripts between FL and LL chickens by either qRT-PCR or RNA-Seq analyses. The FL and LL expression means and their standard errors are provided in S9 Table for genes verified by qRT-PCR analysis.

**Fig 7:**
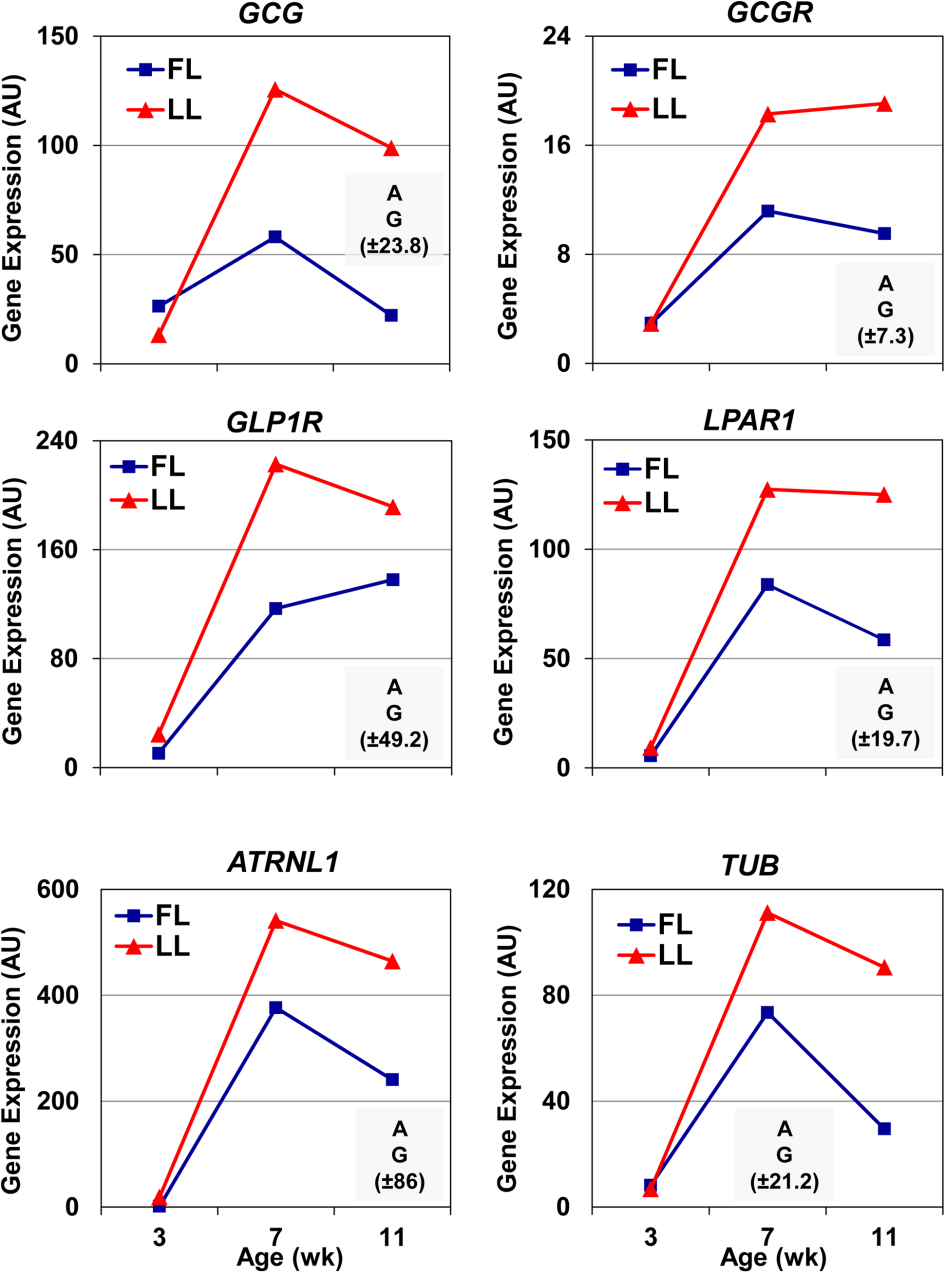
Quantitative RT-PCR analysis of select candidate genes in abdominal fat of FL and LL chickens. Six genes were selected for qRT-PCR analysis of expression profiles in abdominal fat of FL and LL chickens at three ages (3, 7 and 9 weeks of age) to confirm highest expression at 7 wk. Data points represent Least Square Means (LSMEANS; n = 4 birds/genotype) of gene expression in arbitrary units (AU) based on the delta-delta cycle time (^ΔΔ^Ct) method. The general linear models (GLM) procedure in Statistical Analysis System (SAS) software (Cary, NC). A two-factor (genotype x age) analysis of variance (ANOVA) was used to determine significance (*P*≤0.05). The shaded box in each panel indicates significant main effects of genotype (G) or age (A); the number in parenthesis represents the common standard error (SE) of LSMEANS for that gene as determined by the GLM procedure in SAS. *Gene symbols*: attractin-like 1 (*ATRNL1*), glucagon (*GCG*), glucagon receptor (*GCGR*), glucagon-like peptide 1 receptor (*GLP1R*), lysophosphatidic acid receptor 1 (*LPAR1*), and tubby (*TUB*).

The expression of six candidate genes [glucagon (*GCG*), glucagon receptor (*GCGR*), glucagon-like peptide 1 receptor (*GLP1R*), lysophosphatidic acid receptor 1 (*LPAR1*), attractin-like 1 (*ATRNL1*) and tubby (*TUB*)] was initially examined by qRT-PCR analysis at three different ages (3, 7 and 9 wk of age) to verify highest expression at 7 wk (Fig 7). A two-factor analysis of variance [genotype (FL and LL) x age (3, 7 and 11 wk)] shows significant (*P*≤0.05) main effects of genotype and age for all six genes. The abundance of all six genes reached a peak at 7 wk of age and was higher in abdominal fat of the LL than the FL chickens at 7 and 9 wk.

## Discussion

The FL and LL chickens were originally developed by Leclercq *et al* [25] as experimental genetic models to identify the mechanisms controlling abdominal fatness, a complex polygenic trait likely governed by interactions among multiple genes controlling different endocrine and metabolic pathways. Between 7 and 9 wk of age, FL and LL chickens exhibit the greatest difference (2.5- to 2.8-fold) in abdominal fatness while maintaining similar body weights [24,41] and feed intakes [54]. This large divergence in abdominal fatness between FL and LL chickens at 7 wk also corresponds to the highest levels of gene expression in abdominal fat tissue [24]. To maintain a similar body weight despite large differences in abdominal fatness, FL chickens appear to favor partitioning of nutrients (particularly dietary amino acids) into abdominal fat; they also have a higher protein turnover rate [54]. Recent metabolomics studies have clearly demonstrated that the FL and LL chickens are able to maintain their fat and lean phenotypes independent of dietary energy sources, albeit the fatty acid composition of abdominal fat was affected by genotype [32,55]. On the other hand, LL chickens have a greater lean muscle mass and conversely a lower abdominal fat content than do the FL chickens. Several metabolic (liver, adipose tissue, and skeletal muscle) and regulatory (hypothalamus) tissues of the FL and LL chickens have been analyzed using numerous transcriptional methods (i.e., differential mRNA display, qRT-PCR, low- and high-density microarrays, etc.) to unravel the underlying mechanisms of excessive fatness [2,18,24,38,41,42,56,57]. To our knowledge, the present study represents the first deep RNA-Seq analysis of abdominal fat in these unique FL and LL chickens.

Understanding the molecular mechanism of excessive abdominal fatness in the chicken requires examination of differential gene expression using different approaches. Only a few microarray studies have described the abdominal fat transcriptome of chickens [22,58,59]. The most relevant to the present study was the transcriptional analysis of abdominal fat in commercial broiler chickens after a short (5 h) period of acute fasting or immunoneutrilization of insulin [22]. Eighty DE genes identified by RNA-Seq analysis of abdominal fat in FL and LL chickens in the present study were also differentially expressed in visceral fat of broiler chickens subjected to 5 h of fasting [22]. Additionally, five DE genes (*AGTR1*, *AIMP2*, *EEPD1*, *GCG* and *ICA1*) that were identified in abdominal fat after acute insulin immunoneutralization of chickens [22] were also DE genes found presently in FL and LL chickens. Among these genes was *GCG*, the major contra-insulin regulator of glycemia in chickens [60–62], which was expressed higher in abdominal fat of the LL chickens and similar to that observed in chickens after acute insulin immunoneutralization [22]. The endocrine pancreas is the major site of GCG synthesis and secretion in birds [63], while adipose tissue is a major target of GCG, which stimulates lipolysis and release of non-esterified fatty acids into circulation. The GCG receptor, *GCGR*, showed a similar higher expression level in abdominal fat of LL chickens. In agreement with up-regulation of glucagon production and its lipolytic action in abdominal fat of LL chickens, the transcript for the somatostatin receptor (*SSTR2*), which when activated by somatostatin potently inhibits the production of *GCG* [64,65], was highly expressed and up-regulated in FL chickens. Furthermore, *FOXA2*, a transcriptional regulator of differentiation of glucagon producing cells [66], was also up-regulated in visceral fat of the LL chickens. We found that the glucagon-like peptide 1 receptor (*GLP1R*) was nearly 4-fold greater in LL chickens, whereas the glucagon-like peptide 2 receptor (*GLP2R*) was undetectable in abdominal fat of both LL and FL chickens. The absence of *GLP2R* transcripts in our RNA-Seq analysis could be of importance since its expression in adipose tissue of chickens was reported earlier [67]. The presence of *GLP1R* (and absence of *GLP2R*) in FL and LL chickens suggest that differential regulation occurs through the preproglucagon class A transcript (which contains *GCG* and *GLP1*) in abdominal fat of these birds rather than the preproglucagon class B transcript (which contains *GCG*, *GLP1* and *GLP2*) [67]. Furthermore, only the preproglucagon class B transcript was up-regulated in abdominal fat of chickens after insulin immunoneutralization [22]. Our observation of enhanced GCG and GLP1 signaling within abdominal fat of LL chickens supports the idea that visceral fat serves as an important endocrine organ, which displays”ectopic’ expression of several endocrine factors and/or their respective receptors (i.e., *GCG/GCGR*, *GLP1R*, *NPY/NPY2R*, *SSTR2*, *scGH*, *TUB*, *ATRNL1*, *CNR1* and *MC5R)*.

### Altered lipid metabolism in abdominal fat of FL and LL chickens

A recent review provides a comprehensive description of growth and energy metabolism in our divergently-selected FL and LL chickens, amassed from more than three decades of intensive investigation [28]. The “glucose-insulin imbalance” originally described by Simon *et al* [30,31] appears to be the major physiological difference noted between the FL and LL chickens, where the FL exhibit hypoglycemia and only slight hyperinsulinemia, which resembles the ‘pre-obese’ condition of juvenile mammals without true insulin resistance [26]. In fact, the FL chicken appears to be more sensitive to insulin [31] as indicated by their higher rate of glucose utilization and enhanced hepatic lipogenesis [26,35], higher secretion of very-low-density lipoproteins (VLDL) [27], and their greater storage capacity of lipids in abdominal fat [68]. Furthermore, the divergence in abdominal fatness in the FL and LL chickens does not depend on the type of dietary energy, which indicates genetic control [32,55]. Since the liver is considered as the primary site of lipogenesis in birds [10–12], most previous studies of the FL and LL were directed at understanding the genetic difference in hepatic lipogenesis, rather than the lipogenic capacity of abdominal fat.

A major finding of our time course microarray analysis of abdominal fat in FL and LL chickens [24] was the discovery of numerous DE genes involved in lipid synthesis. We have confirmed the up-regulation of many DE lipogenic genes in abdominal fat of FL chickens by both RNA-Seq and qRT-PCR analyses. Further, the abundant expression of lipogenic genes in abdominal fat in the FL was validated by the report that acute fasting [22] depressed expression of several lipogenic genes in the chicken. For example, the lipogenic enzyme *ME1*, which is critical for the synthesis of lipids via generation of NADPH required for the conversion of malonyl-CoA to palmitic acid (by *FASN*), was down-regulated in LL chickens and similar to its lower expression in adipose tissue of fasted chickens. Both the soluble (*MDH1*) and mitochondrial (*MDH2*) forms of malate dehydrogenase, which are expressed higher in adipose tissue and liver [57] of FL chickens, were among the top six percent of the highest expressed genes found by RNA-Seq analysis of abdominal fat in FL and LL chickens at 7 wk. Similar to the FL chickens [68], the expression of *MDH1*, *MDH2* and *ME1* show a strong positive correlation with increased adipocyte volume in genetically fat pigs [69]. Another gene differentially expressed in response to either genetic or nutritional perturbation is lipin 1 (*LPIN1*), which was identified as a reciprocal regulator of triglyceride synthesis and hydrolysis in adipocytes of *LPIN1* knockout mice [70]. In chickens, adipose *LPIN1* expression is elevated in response to short-term (5 hr) food deprivation [22], a 30% energy restriction [71] or in “leaner” and slow-growing Leghorn and Fayoumi breeds when compared to “fatter” broiler chickens [23]. However, *LPIN1* was expressed higher in abdominal fat of our genetically fat (FL) chickens as indicated by both RNA-Seq and qRT-PCR analyses (see Fig 6B). The present study clearly shows that divergent genetic selection of broiler chickens for a 2.5–2.8 fold difference in abdominal fatness results in higher expression of *LPIN1* in visceral fat of the FL, but not the LL chickens. Of our cross-validated candidate genes, *LPIN1* is of particular interest for its potential to control several important metabolic processes including adipogenesis, lipogenesis and eicosanoid signaling. Involvement of *LPIN1* in the regulation of adipogenesis occurs via control of phosphatidic acids levels, which influence *PPARG* expression [72] and LPIN1 is required for PPARG-driven adipogenesis both *in vitro* and *in vivo* [73,74]. Further, LPIN1 is also a co-regulator of nuclear receptor signaling by interaction with the PPARs (PPARA, PPARG and PPARGC1A) and CEBPA [75,76]. We found that atrial natriuretic peptide receptor 3 (*NPR3*) and AMP-activated protein kinase (AMPK) were both up-regulated in abdominal fat of the FL chickens (see Table 4). Atrial natriuretic peptide (ANP) provokes a strong lipolytic action in human adipocytes by activating AMPK and increasing mitochondrial biogenesis [77].

In the present study, we found that the expression of *KLF5* was 2.5-fold greater in abdominal fat of the LL than in the FL at 7 wk (Table 3; S8 Table). The transcription factor *KLF5* has been implicated in adipogenesis, since knockout of *KLF5* retards expansion of white (visceral) adipose tissue [78]. Furthermore, this study shows that over-expression of *KLF5* stimulates adipocyte differentiation via interactions with *CEBPB* and *PPARG*, which are key regulators of adipocyte differentiation. A recent study of *KLF2* expression in abdominal fat during juvenile development (1–12 wk) of a different population of divergently-selected FL and LL broiler chickens shows that the abundance of *KLF2* was greater in the LL at 1 wk, but higher in fat of the FL between 3–8 wk [79]. Functional *in vitro* analyses indicate that over-expression of *KLF2* inhibits preadipocyte differentiation and expression of *CEBPA* and *PPARG*. These observations support their conclusion that *KLF2* plays a role in divergent genetic selection of this distinct population of FL and LL chickens. Clearly, the role of multiple transcription factors in the divergence of abdominal fat accretion in FL and LL chickens needs further investigation.

Hormone signaling appears to play a major role in controlling lipid metabolism in abdominal fat of FL and LL chickens. For example, growth hormone (GH) signaling is highly active in abdominal fat of the LL birds (see Fig 2B). We found that the peripheral receptor for melanocyte-stimulating hormone and adrenocorticotropic hormone, *MC5R*, was expressed higher in abdominal fat of FL chickens. MC5R regulates fatty acid oxidation in skeletal muscle of mice [80] and in hepatocytes of sea bass, where a MC5R agonist stimulates lipolysis and the release of free fatty acids from cultured sea bass adipocytes [81]. Furthermore, MC5R has been associated with obese phenotypes in humans [82]; and the *MC5R* gene locus is highly conserved in the genomes of human, mouse and chicken [83]. Additionally, the membrane-bound progesterone receptor (*PGRMC1*) was highly expressed in abdominal fat of FL chickens (see Table 4). The PGRMC1 directly regulates cholesterol synthesis and metabolism of steroid hormones in mammals [84]. Progesterone signaling seems to be enhanced in abdominal fat of LL chickens as indicated by the 2-fold up-regulation of the progesterone receptor (*PGR*), which regulates the expression of 25 DE target genes in visceral fat. Progesterone administration in rats increases body and inguinal fat mass, a response only observed in females [85], whereas the significance of up-regulation of *PGR* in abdominal fat of LL cockerels in the present study remains unknown.

Androgen signaling also appears to be up-regulated in abdominal fat of LL chickens, where the *AR* was expressed 40 percent higher compared to their fatter counterparts. Androgen signaling in adipose tissue of murine models protects against obesity and regulates insulin action and glucose homeostasis, and *AR* knockout mice are more susceptible to high-fat diet–induced visceral obesity [86]. The up-regulation of *AR* and the 46 DE genes that are direct targets of AR found presently in abdominal fat of the LL suggests a similar mechanism for *AR* signaling in adipose tissue of chickens. Unfortunately, a recent study of gene expression in castrated (capon) chickens did not include a transcriptional analysis of abdominal fat in the capons, which become fatter, nor in the leaner intact or testosterone-implanted groups [87]. This report clearly shows the importance of androgen in limiting excessive fattening in the chicken, albeit more attention was focused on over-expression of *PCK1* in the liver of capons rather than in abdominal fat as a direct target of androgen signaling.

Another intriguing finding in abdominal fat of FL and LL chickens was the abundance of genes involved in eicosanoid signaling. This includes production of eicosanoid precursors as well as downstream signaling events. In human breast cancer MCF7 cells, loss of FADS2 function blocks normal polyunsaturated fatty acid biosynthesis resulting in the FADS1 generation of polyunsaturated fatty acids which lack the 8–9 double bond of eicosanoid signaling precursors (i.e., arachidonic acid and eicosapentaenoic acid) [88]. Both *FADS2* (higher in FL chickens) and *FADS1* (higher in LL chickens) are differentially expressed in the present study. Another enzyme involved in eicosanoid signaling, *DAGLA* (higher in LL chickens), catalyzes the hydrolysis of diacylglycerol (DAG). The product of this reaction is arachidonic acid precursor 2-arachidonoyl-glycerol (2-AG), a major peripheral endocannabinoid signaling molecule [89]. In adipose tissue of rodents, 2-AG activates the CNR1 (a gene also up-regulated in LL chickens), an event that is up-regulated in fat treatment groups [89–91]. Furthermore, *FAAH*, the enzyme that degrades the active endocannabinoid 2-AG [92] was up-regulated in abdominal fat of FL chickens. Taken together, these findings provide evidence for enhanced endocannabinoid signaling in adipose tissue of the LL chickens.

### Hemostatic gene expression in abdominal fat of chickens

**Fig 8:**
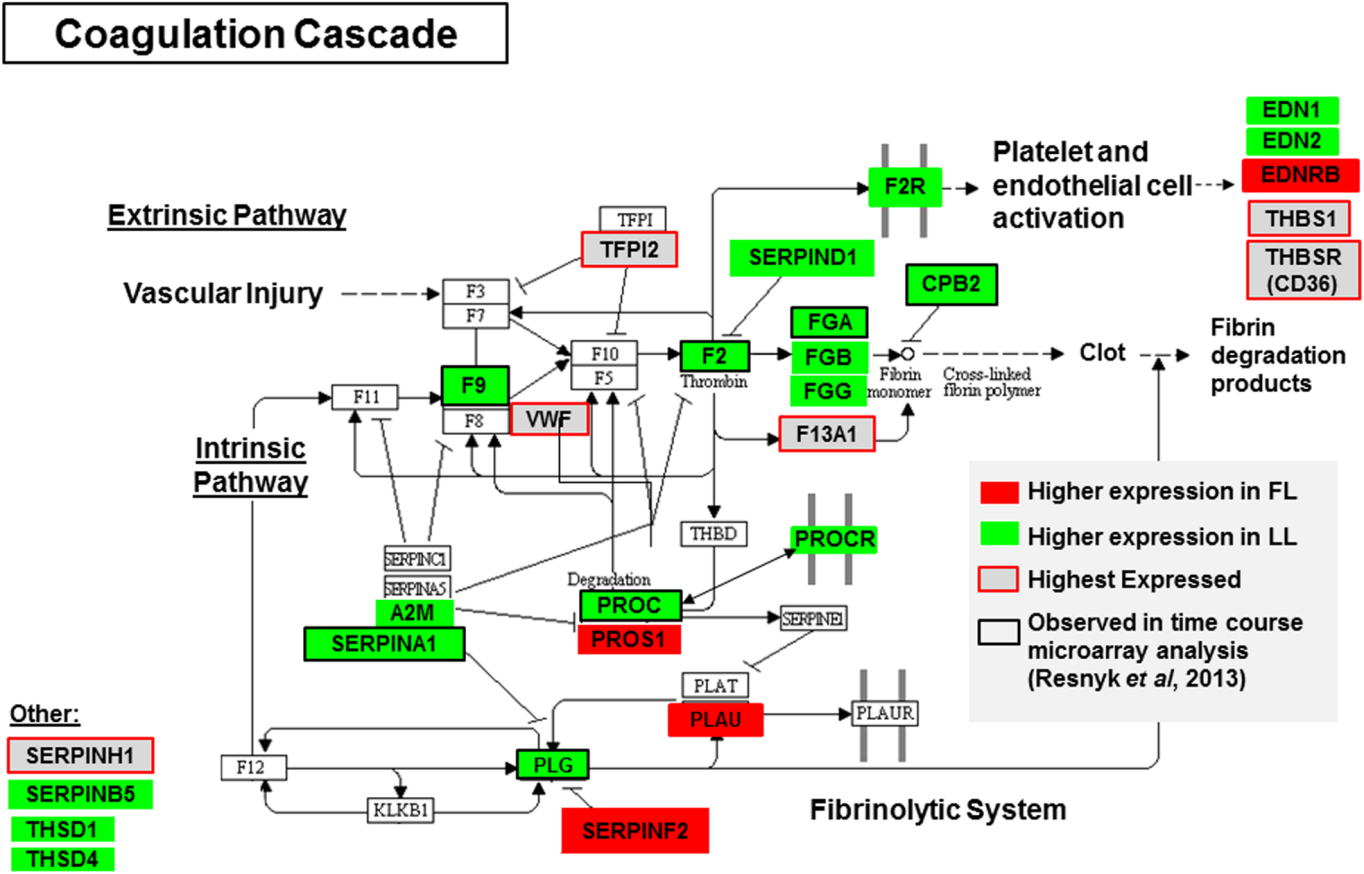
Prevalence of coagulation factors in abdominal fat transcriptome of FL and LL chickens. This coagulation cascade was adapted from the Complement and Coagulation Cascades (map04610) found in the Kyoto Encyclopedia of Genes and Genomes (KEGG). Reprinted from the KEGG website (http://www.genome.jp/kegg-bin/show_pathway?map04610] under a CC BY license, with permission from KEGG, original copyright [map04610, Kanehisa Laboratories, April 03, 2009]. Genes are colored based on differential gene expression (FL/LL) levels from the present RNA-Seq analysis at 7 wk and our recent time-course (1–11 wk) microarray study [24] of abdominal fat in FL and LL chickens. The highest-expressed coagulation genes from the RNA-Seq analysis are also shown, along with several other genes related to hemostasis and angiogenesis.

The predominant expression of numerous hemostatic genes in abdominal fat of FL and LL chickens is clearly illustrated by an overlay on the coagulation cascade from the Kyoto Encyclopedia of Genes and Genomes (KEGG; http://www.genome.jp/kegg/) website (Fig 8). Seven hemostatic genes, up-regulated in the LL, were identified earlier in our time-course (1–11 wk) microarray analysis of abdominal fat in the FL and LL chickens [24]. The present RNA-Seq analysis of abdominal fat in the FL and LL chickens at 7 wk of age verifies and extends the remarkable involvement of the hemostatic system in divergent genetic selection of abdominal fatness in meat-type chickens. From our RNA-Seq analysis, an additional 11 genes [*A2M*, *END1*, *END2*, *FGB*, *FGG*, *F2R*, protein C receptor, endothelial *(PROCR)*, *SERPIND1*, *SERPINB5*, *THSD1*, and *THSD4*] were up-regulated in the LL chickens, whereas another 4 genes (*PROS1*, *PLAU*, *SERPINF2* and *EDNRB*) were expressed at higher levels in visceral fat of the FL at 7 wk. The extraordinary involvement of the coagulation system in limiting expansion of fat mass in the LL chickens is further demonstrated by the high abundance of additional six hemostasis-related genes (*VFW*, *TFPI2*, *F13A1*, *SERPINH1*, *THBS1* and *THBSR*) across both genotypes. Furthermore, our time-course microarray analysis of liver samples from the same FL and LL chickens used for transcriptional analyses of their abdominal fat failed to show any effects of the FL or LL genotype on hepatic expression of these coagulation factors [Unpublished work; see NCBI GEO accession GSE8812]. Interestingly, the coagulation factor XIII, A1 polypeptide (*F13A1*) was previously identified as a novel obesity candidate gene in humans [93]. Collectively, our previous time-course microarray analysis [24] and the present RNA-Seq analysis of abdominal fat in the FL and LL chickens at 7 wk support the idea that coagulation and angiogenic factors are critical components in divergent genetic selection of visceral fat mass, which seem to function locally (paracrine or autocrine) and independently of the hemostatic system found in systemic circulation, since the liver is the main source of coagulation factors found in the bloodstream. The recent RNA-Seq analysis of breast muscle from broiler-type chickens afflicted with “Wooden Breast disease” has implicated the chicken’s coagulation system in progression of this novel muscular disorder [48]. Birds suffering from this myopathy exhibit down-regulation of eight hemostatic genes (*A2M*, *FGA*, *FGB*, *FGG*, *KNG1*, *SERPINC1* and *VWF*) and up-regulation of four other coagulation genes (*F5*, *F10*, *PROS1* and *F2R*).

The serine protease thrombin (*F2*), which we identified as a DE gene in the time course microarray analysis of abdominal fat of the FL and LL chickens [24], is an extremely potent platelet agonist. In the present RNA-Seq study at 7 wk, *F2* was not significantly different (1.3-fold higher in LL chickens; *P*≤ 0.07; data not shown) between the genotypes. Interestingly, we found that the receptor for *F2* [*F2R* or proteinase-activated receptor–1 (*PAR1*)] was expressed higher in adipose tissue of LL chickens. PAR1 and PAR4 are expressed in human adipose tissue, where treatment with F2 induces expression and secretion of several adipokines (IL-1B, IL–6, MCP–1, and VEGF) [94]; however, this is mediated through the PAR4 rather than PAR1. In another study, Kajimoto *et al*. [95] found that the interaction of F2 and PAR1 in 3T3-L1 adipocytes stimulates FABP4 which regulates the expression of interleukin 6 (IL–6) and vascular endothelial growth factor (VEGF). Although there have been no studies describing F2-PAR1 interactions in adipose tissue of chickens, an earlier report indicates that interaction between thrombin and PAR1 is required for “endothelial mesenchymal transdifferentation” in the aorta of chick embryos [96]. Our present study shows that genes involved in thrombin signaling are differentially expressed in visceral fat of FL and LL chickens. The function of these coagulation factors in regulating expansion of visceral fat mass in the chicken certainly needs further definitive investigation.

The up-regulation of numerous genes involved in hemostasis (pro-coagulation and anti-coagulation) in visceral fat of LL chickens was originally reported in our time-course microarray analysis [24]. Although prothrombotic genes are associated with increased fatness in humans [97–100], our studies suggest a novel role of several hemostatic factors in limiting abdominal fat accretion in the LL chickens [24]. The present RNA-Seq analysis (at 7 wk) and our previous time-course (1–11 wk) microarray study [24] clearly show differential expression of numerous pro- and anti-coagulation genes in abdominal fat of FL and LL chickens (see Fig 8). Most remarkably, abdominal fat of LL chickens appears to be in a pro-coagulation state, since five genes driving thrombosis are up-regulated (*F9*, *F2*, *FGA*, *FGB*, and *FGG*). RNA-Seq analysis identified seven additional genes (*F2R*, *FGB*, *FGG*, *PROCR*, *PROS1*, *PLAU*, and *SERPINF2*) that were not found by our previous microarray analysis. Four related genes [serpin peptidase inhibitor, clade H, member 1, (*SERPINH1*), serpin peptidase inhibitor, clade B, member 5 (*SERPINB5*), thrombospondin, type I, domain containing 1 (*THSD1*) and thrombospondin, type I, domain containing 4 (*THSD4*)] were also identified by RNA-Seq analysis of abdominal fat. Likewise, five additional genes [endothelin 1 (*EDN1*), endothelin 2 (*EDN2*), endothelin receptor type B (*EDNRB*), thrombospondin 1 (*THBS1*) and thrombospondin receptor (*CD36*)] were found in the present study; these genes are usually involved in vasoregulation or activation of platelets and endothelial cells. We propose that these proteases and protease inhibitors found in visceral fat of the chicken could control the proteolytic activation or deactivation of adipokines and other endocrine factors. The down-regulation of *PROS1*, an inhibitor of blood coagulation, in the LL supports a pro-coagulation state in abdominal fat of these chickens. Further, the higher expression of *PROC* [24] and the PROC receptor (*PROCR*) in visceral fat of the LL chickens suggests increased activation of PROC and PAR1 (or F2R) by the multifunctional PROCR [101]. Fibrinogen (*FGA*, *FGB* and *FGG*) is mainly produced in the liver and secreted into systemic circulation. Interestingly, fibrinogen expression in FL chickens is nearly undetectable whereas all three fibrinogen subunits are expressed 10-fold higher in visceral fat of the LL. Similarly, the level of FGG in plasma was inversely related to adiposity in subcutaneous and visceral fat in humans [102] and in rats fed a high-fat diet for 8 wk [103]. Aside from its primary function in blood coagulation, fibrinogen has been identified as a binding surface for several proteins involved in vascular homeostasis [104]. The over-expression of fibrinogen and albumin (*ALB*) in abdominal fat of LL chicken could contribute to increased binding, uptake and/or transport of adipose-derived proteins. The differential expression of the large number of hemostatic factors observed in abdominal fat, but not in liver of our FL and LL chickens was not observed during nutritional or hormonal perturbation in the chicken [22], which suggests that these coagulation genes could have a novel role in divergent selection and the 2.5-fold difference in abdominal fatness observed in our unique experimental lines.

These hemostatic factors could contribute to local (paracrine/autocrine) and/or endocrine control of fat accretion and/or vasomotor tone, which would have important consequences on adipogenesis [105], angiogenesis and regulation of blood flow [106]. This apparent vascular regulation in visceral fat seems highly complex and under control of several mechanisms. One such mechanism is the renin-angiotensin system (RAS), which usually serves as a major systemic regulator of blood pressure; now, it is known that many components of RAS are also expressed in adipose tissue [107]. Support for this idea is provided by the present study and our observation that 11 of the 13 DE genes found in the renin-angiotensin signaling pathway, are expressed higher in abdominal fat of the LL chickens. As an active RAS ligand, angiotensin II is involved in lipid metabolism via regulation of insulin signaling in adipose tissue [105,108]. RAS plays an important role in the regulation of local and systemic blood pressure via vasoconstriction of peripheral arterioles. The G-coupled protein receptor for angiotensin II, *AGTR1*, was up-regulated in chickens after insulin immunoneutralization or acute fasting [22]. In the present study, we found that both *AGTR1* and *AGTR2* were down-regulated in adipose tissue of FL chickens (see Table 4). The down-regulation of angiotensin II receptors in adipose tissue of anabolic chicken models (FL, fed or normal insulin activity) suggests increased vasodilation in abdominal fat, a process that accompanies angiogenesis [109], allowing for increased blood flow and expansion of adipose mass. Furthermore, an overactive RAS could inhibit adipocyte differentiation [105] resulting in down regulation of adipogenesis in LL chickens.

Vascular tone is also mediated through the release of vasoconstrictors or vasodilators independent of the RAS, including catecholamines, endothelins and nitric oxide. Reduced expression of potent vasoconstrictors (*EDN1* and *EDN2*) in adipose tissue of FL chickens strengthens the importance of increased vasodilation and therefore enhanced blood flow to support higher accretion of abdominal fat in FL chickens. While the endothelin receptor B (*EDNRB*) transcript was slightly higher in FL chickens (1.5-fold), the abundance of *EDNRA* was more than 10-fold higher, which suggests that EDNRA is the more active isoform in adipose tissue of FL chickens. In mammals, the major effects of vasodilation are mediated through either down regulation of vasoconstrictors or increased generation of nitric oxide (NO). The production of NO by nitric oxide synthase (NOS) is usually driven by hypoxia [110], where hypoxic conditions in adipose tissue usually lead to overproduction of and ultimately resistance to NO [111]. Since *HIF1A* was down-regulated in visceral fat of FL chickens and the abundance of *NOS* similar between genotypes, hypoxia and over-production of NO do not seem to be an issue. Rather, regulation of NO signaling in these chickens could be controlled by phosphodiesterases, which are expressed at much lower levels in FL chickens (*PDE1C*, *PDE3A*, *PDE5A*, *PDE9A* and *PDE10A*). Phosphodiesterases catalyze the degradation of cGMP [112], ultimately inhibiting the vasodilatory action of NO, and have been implicated in the treatment of cardiovascular diseases [113], respiratory diseases [114] and metabolic syndrome [115]. For example, PDE1C, a non-selective phosphodiesterase, is amongst the highest expressed phosphodiesterases in rat β-islet cells and knockdown of this gene significantly increases insulin secretion [116]. Higher expression of another nonselective phosphodiesterase, *PDE3A*, in adipose tissue in humans was correlated with weight loss after gastric bypass surgery [117]. Furthermore, inhibition of cGMP-specific phosphodiesterases (i.e., PDE5A and PDE9A) is effective in cardio-protection [113]. Taken together, altered RAS and cGMP signaling within adipose tissue of the FL chickens could ultimately result in vasodilation and increased blood flow to support their higher fat deposition.

The up-regulation of growth factors (*PDGFC* and *FGFR3*; see Fig 6A) and *GREM1* in abdominal fat of FL chickens could support enhanced angiogenesis. The higher expression of *FGFR3* in FL chickens is similar to that observed in adipose tissue of *ApoE* knockout mice, which exhibit increased adiposity when fed a high fat diet [118]. The expression of *GREM1*, a bone morphogenetic protein antagonist, correlates with increased angiogenesis in humans with pancreatic neuroendocrine tumors [119]; whereas, the knock-down of *GREM1* in human HK–2 cells increases *BMP7* signaling activity [120]. Correspondingly, *BMP5* and *BMP7* were down- regulated in abdominal fat of the FL chickens, whereas *BMP15* expression was strongly up-regulated in these birds. The expression of *BMP5* is down-regulated under highly vascularized conditions, like those simulated by tumor cell lines (i.e., adrenocortical carcinoma and adrenocortical tumor cell lines [121]). Another angiogenesis-related gene endoglin (*ENG*), a co-receptor for TGFβ, was expressed higher in abdominal fat of the LL chickens (see Fig 3B and Table 4). Knockout of this gene (*Eng* ^*-/-*^) is lethal to mid-gestation mouse embryos due to severely impaired angiogenesis and cardiac development [122,123]. Further, plasma insulin and hepatic triglyceride levels are reduced in heterozygous *Eng* ^*+/-*^ mice fed a high-fat diet [124]. In addition, NPY-stimulated angiogenesis and adipogenesis in the mouse are mediated through the NPY2R receptor, a pathway augmented by glucocorticoids [125]. This study using mice provides strong evidence that up-regulation of NPY secretion and *NPY2R* expression within visceral fat contributes to obesity and metabolic syndrome in mammals. Our qRT-PCR analysis clearly shows overexpression of both *NPY* and *NPY2R* in visceral fat of FL chickens, which could contribute to their enhanced adipogenesis and angiogenesis. This idea is supported by a recent report of higher expression of *NPY* and its receptors (*NPY1R* and *NPY5R*) in abdominal fat of fatter high-growth chickens than that of the slow-growing leaner chickens [126]. Furthermore, NPY treatment of cultured chicken adipocytes boosts NPY2R-mediated proliferation, adipogenesis and lipid accumulation, which implicate a functional role for adipose-derived NPY and the NPY2R in expansion of visceral fat in the chicken [127]. The combined over-expression of angiogenic genes and under-expression of vasoactive factors identified in the present study could promote increased blood flow to support enhanced growth of visceral adipose tissue in FL chickens.

Collectively, our previous time-course microarray study [24] and the present RNA-Seq analysis of abdominal fat in FL and LL chickens have identified several novel features of the transcriptional response to divergent genetic selection for extremes in visceral fatness. The assumption that avian and mammalian species share similar mechanisms that regulate energy metabolism and adipogenesis must be tempered with a heightened awareness of novel avian characteristics. Namely, the loss of multiple adipokine genes (*LEP* [7], *PAI–1*, *TNFA*, resistin and omentin [8]) from the chicken genome has a major impact on our understanding of the avian regulatory systems that control energy intake, metabolism and adiposity. Most discussions of insulin resistance, type–2 diabetes, inflammation, and obesity in mammals are centered on involvement of these major adipokines. Many of the unique aspects of metabolism, including fasting hyperglycemia and insulin insensitivity, are likely reflected by absence of these and perhaps other “mammalian” regulators in the chicken—the premier avian model [1–4].

In summary, our RNA-Seq analysis of visceral adipose tissue in meat-type chickens divergently selected for a large difference in abdominal fatness reveals higher expression of numerous hemostatic and lipolytic genes in genetically lean chickens, whereas abundant expression of lipogenic, angiogenic and adipogenic genes were found in fat chickens. In both genotypes, abdominal fat shows “ectopic” expression of numerous endocrine factors and/or receptors that could contribute to their divergence in visceral fatness. We propose that over-expression of multiple hemostatic genes encoding serine proteases and serine protease inhibitors in visceral fat of lean chickens could be involved in processing of adipokines and other endocrine factors. The present RNA-Seq analysis of abdominal fat in genetically fat and lean chickens provides strong support for the idea that adipose tissue is an important endocrine organ in chickens. In addition, the abundance and differential expression of several key lipogenic and adipogenic genes in genetically fat chickens suggests that abdominal fat could make a more important contribution to lipid synthesis in birds than previously recognized. This idea is supported by an earlier paper [128], which indicated that fatty acids synthetized in liver of chickens are subject to oxidation by liver and muscle, whereas fatty acids synthetized by abdominal fat are retained there for storage. The importance of functional candidate genes identified in visceral fat from the present transcriptional profiling and bioinformatics analyses will require further definitive studies to verify their importance in lipogenesis and adipogenesis, and to gain mechanistic insight to genetic control of adiposity in the chicken.

## Supporting Information

S1 Fig Power analysis of the FL and LL abdominal fat RNA-Seq dataset. (**A**) A power analysis was conducted to demonstrate adequate biological samples size using the web-based software program called “Scotty” (http://euler.bc.edu/marthlab/scotty/scotty.php). Using the average of 38.5 million reads per sample, the power was calculated at ≥1.5, 2, or 3-fold change (-X change) differences between FL and LL chickens at a significance of *P*≤0.01. We achieved the power to detect 80% genes with ≥1.5-fold differences as indicated by the red broken line. (**B**) The “Scotty” program also performed a hierarchical cluster analysis using the Spearman correlation as the distance metric to demonstrate relatedness among the eight individual (4 FL and 4 LL) birds used in the RNA-Seq analysis.
(TIF)

S1 Table Information on design of primers used for qRT-PCR analysis. For each primer used for qRT-PCR analysis, gene symbol, gene name, forward and reverse primer sequences, GenBank accession, and amplicon size (bp) are provided. Primers were designed from indicated NCBI GenBank accessions using Primer Express 2.1 software (Applied Biosystems, Inc.).
(XLSX)

S2 Table Detailed summary of RNA-Seq analysis. This table provides a summary for all samples used in the RNA-Seq analysis. Information for each biological sample includes: multiplexing number, genotype, NCBI GEO sample ID, total input reads, single reads after trimming, paired-end reads after trimming, total reads mapped, single reads mapped, paired-end reads mapped, total reads unmapped, single reads unmapped, paired-end reads unmapped, genes with reads per kilobase of transcript per million mapped reads (RPKM) > 0, and transcripts with RPKM > 0.
(XLSX)

S3 Table Highest expressed (HE) genes in abdominal fat of FL and LL chickens at 7 wk. The table contains a list of the top 5% of highly-expressed genes found in adipose tissue. Information provided for each gene includes feature ID, gene symbol, Entrez Gene name, fold change and average reads (i.e., number reads averaged across FL and LL chickens for each gene). No fold-difference or statistical cutoff was applied to the Highest Expressed (HE) Gene list (Worksheet 1). Two additional worksheets provide a list of HE genes with a < ±1.2-fold difference in FL/LL expression ratio [164 HE genes in FL (>1.2-fold difference; Worksheet 2) and 155 HE genes in LL (< -1.2-fold difference; Worksheet 3).
(XLSX)

S4 Table Differentially expressed (DE) genes in abdominal fat between FL and LL chickens at 7 wk. This table provides the feature ID, gene symbol, gene description, FL normalized means, LL normalized means, Fold-change (±1.2-fold difference), FDR corrected p-value (*P*≤ 0.05), chromosome, strand, Start Position, End Position, UniProt Accession (UniProAC) and Protein Family (Pfam) Description for the differentially-expressed (DE) genes from the RNA-Seq analysis.
(XLSX)

S5 Table DE genes in abdominal fat of FL and LL Cockerels associated with lipid metabolism. This table contains 607 genes that are known to be involved in lipogenesis or lipolysis according to their annotation in the Ingenuity Knowledge Base. The “Average reads” column contains the number of reads from the RNA-Seq analysis of abdominal fat for that gene averaged across FL and LL genotypes.
(XLSX)

S6 Table Top canonical pathways highly populated by DE genes from RNA-Seq analysis of abdominal fat in FL and LL cockerels at 7 wk. This Excel file contains five worksheets that list DE genes that populate the five top canonical pathways identified by IPA analysis (see Table 3). The top five canonical pathways from IPA analysis are: “Adipogenesis Pathway, Glucorticoid Receptor Signaling, Axonal Guidance Signaling, Hepatic Fibrosis, and RAR Activation”. Each worksheet lists the following information: gene symbol, Entrez gene name, fold-change (FL/LL), cellular location, and type of molecule.
(XLSX)

S7 Table Top toxicology (tox) functions identified by IPA analysis of DE genes in abdominal fat of FL and LL cockerels at 7 wk. This Excel file contains five worksheets of DE genes that populate the five toxicology functions identified by IPA analysis (see Table 2). The top five toxicology functions from IPA analysis are: “Cardiac Hypertrophy, RAR Activation, Liver Proliferation, Oxidative Stress, and TGF-β Signaling”. Each worksheet lists the following information: gene symbol, Entrez gene name, fold-change (FL/LL), cellular location and type of molecule.
(XLSX)

S8 Table Sixteen DE genes identified by Ingenuity Upstream Regulator Analysis are known transcription regulators. This Excel file contains a worksheet that list 16 DE genes identified by IPA as “Upstream regulators”. The worksheet list the following information: gene symbol, fold-change (FL/LL), function of regulator, IPA activation z-score, P-value of overlap, the number of target genes, and list of all direct target genes of that transcription regulator in the DE dataset from RNA-Seq analysis.
(XLSX)

S9 Table Verification of gene expression from RNA-Seq of abdominal fat in FL and LL cockerels by qRT-PCR analysis. This Excel file contains a worksheet that lists the genes used for qRT-PCR verification of differential gene expression, the average expression level ±SEM, and the significance level between four FL and 4 LL cockerels (see Table 5 for FL and LL means ±SEM).
(XLSX)

## Abbreviations

FL: Fat line
LL: Lean line
DE: differentially expressed
HE: highest expressed
wk: week of age
IPA: Ingenuity Pathway Analysis
qRT-PCR: quantitative real-time polymerase chain reaction
h: hour(s)
