# Supplementary figures and images for: RNA-Seq Analysis of Abdominal Fat in Genetically Fat and Lean Chickens Highlights a Divergence in Expression of Genes Controlling Adiposity, Hemostasis, and Lipid Metabolism
Source: PLoS One. 2015 Oct 7;10(10):e0139549. doi: 10.1371/journal.pone.0139549 (PMC4596860; doi:10.1371/journal.pone.0139549)

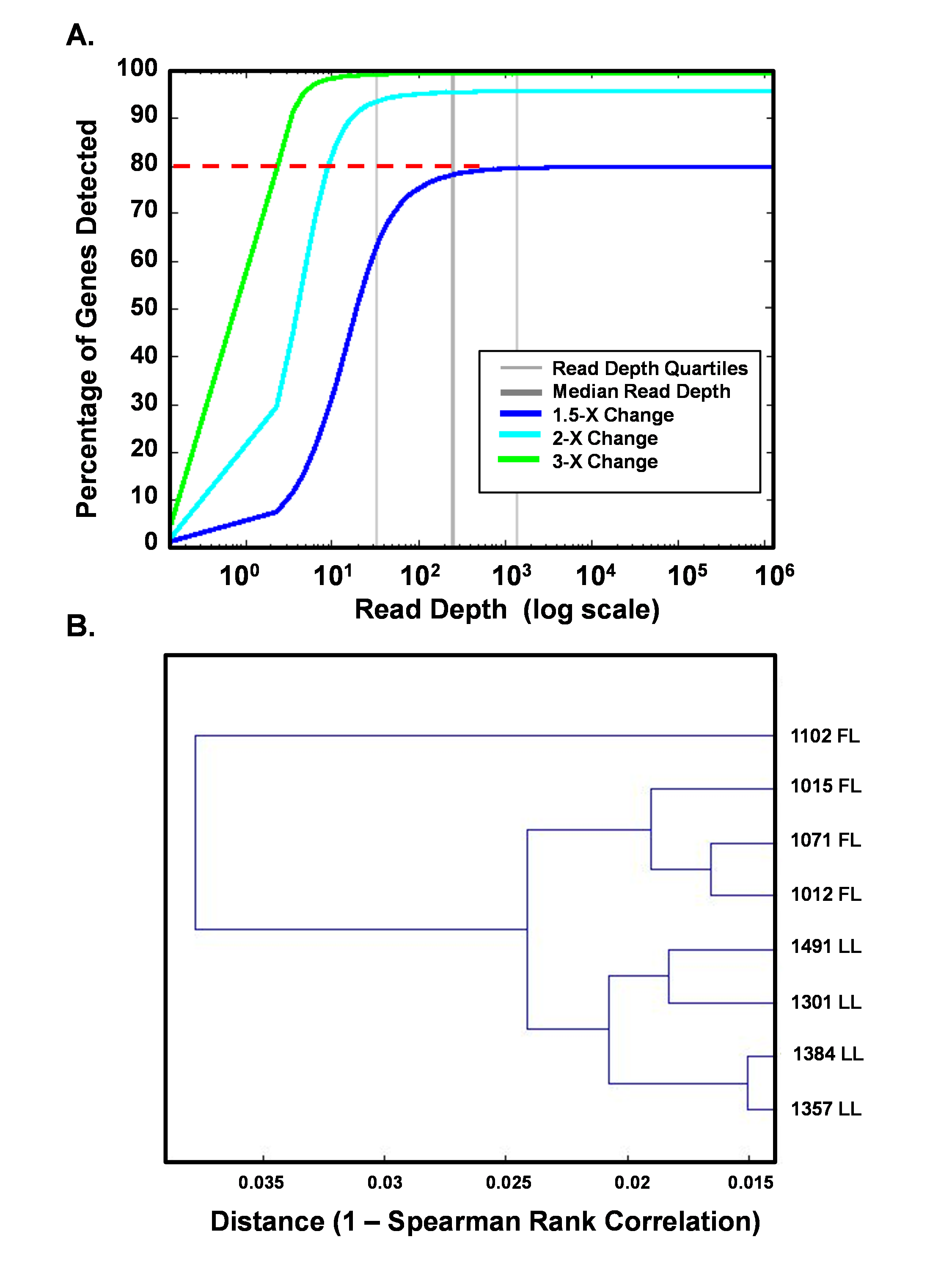

Supplement: S1 Fig — (A) A power analysis was conducted to demonstrate adequate biological samples size using the web-based software program called “Scotty” (http://euler.bc.edu/marthlab/scotty/scotty.php). Using the average of 38.5 million reads per sample, the power was calculated at ≥1.5, 2, or 3-fold change (-X change) differences between FL and LL chickens at a significance of P≤0.01. We achieved the power to detect 80% genes with ≥1.5-fold differences as indicated by the red broken line. (B) The “Scotty” program also performed a hierarchical cluster analysis using the Spearman correlation as the distance metric to demonstrate relatedness among the eight individual (4 FL and 4 LL) birds used in the RNA-Seq analysis. (TIF) [file pone.0139549.s001.tif]
